# Cell proliferation and Notch signaling coordinate the formation of epithelial folds in the *Drosophila* leg

**DOI:** 10.1242/dev.202384

**Published:** 2024-04-16

**Authors:** Alonso Rodríguez, David Foronda, Sergio Córdoba, Daniel Felipe-Cordero, Antonio Baonza, David G. Miguez, Carlos Estella

**Affiliations:** ^1^ Centro de Biología Molecular Severo Ochoa (CSIC-UAM), Universidad Autónoma de Madrid, Madrid 28049, Spain; ^2^ Departamento de Medicina, Facultad de Ciencias Biomédicas y de la Salud, Universidad Europea de Madrid, Madrid 28670, Spain; ^3^ Departmento de Física de la Materia Condensada, Instituto de Física de la Materia Condensada (IFIMAC), Facultad de Ciencias, Universidad Autónoma de Madrid, Madrid 28049, Spain

**Keywords:** *Drosophila*, Notch, Cell proliferation, Epithelial folding, Morphogenesis, Tissue compression

## Abstract

The formation of complex three-dimensional organs during development requires precise coordination between patterning networks and mechanical forces. In particular, tissue folding is a crucial process that relies on a combination of local and tissue-wide mechanical forces. Here, we investigate the contribution of cell proliferation to epithelial morphogenesis using the *Drosophila* leg tarsal folds as a model. We reveal that tissue-wide compression forces generated by cell proliferation, in coordination with the Notch signaling pathway, are essential for the formation of epithelial folds in precise locations along the proximo-distal axis of the leg. As cell numbers increase, compressive stresses arise, promoting the folding of the epithelium and reinforcing the apical constriction of invaginating cells. Additionally, the Notch target *dysfusion* plays a key function specifying the location of the folds, through the apical accumulation of F-actin and the apico-basal shortening of invaginating cells. These findings provide new insights into the intricate mechanisms involved in epithelial morphogenesis, highlighting the crucial role of tissue-wide forces in shaping a three-dimensional organ in a reproducible manner.

## Teaser

**Summary:** This study investigates how the intricate interplay between proliferation and patterning mechanisms enables the precise and reproducible sculpting of three-dimensional organs.

## INTRODUCTION

The transition during development from a relatively flat epithelium to a complex three-dimensional structure largely relies on tissue folding during morphogenesis. The precise location of the folds that shape an organ is dictated by the underlying genetic patterning network, and changes in cell shape and mechanical forces, such as tension and compression, drive the process (Zartman and Shvartsman, 2010). Asymmetrical mechanical forces generated at both local and tissue-wide levels are the main inductors of the cell shape changes that promote epithelial folding (Tozluoglu and Mao, 2020). Different mechanisms, including apical constriction (Martin and Goldstein, 2014), apoptotic basal extrusion (Monier et al., 2015), mitotic cell rounding (Kondo and Hayashi, 2013), or basal relaxation and lateral tension (Sui et al., 2018) contribute to the generation of these local forces. In addition, differential growth between tissue layers or within the tissue plane may also contribute to tissue folding (Tozluoglu et al., 2019; Trushko et al., 2020; Savin et al., 2011; reviewed by Tozluoglu and Mao, 2020). An important question is how are these local and tissue-wide mechanisms coordinated to drive epithelial folding in a highly reproducible pattern?

*Drosophila* imaginal discs provide an excellent model to study morphogenesis due to their stereotyped and reproducible epithelium folding, coupled with a well-established understanding of the underlying patterning mechanisms (Ruiz-Losada et al., 2018). Imaginal discs are epithelial sac-like structures formed by a monolayer of pseudostratified cells that, during larval development, grow and become progressively subdivided in territories with different cellular identities. At metamorphosis, imaginal discs give rise to adult external structures such as the wing or the leg (Ruiz-Losada et al., 2018). The leg disc is segmented along the proximo-distal axis, where each segment is defined by a distinct code of transcription factors that dictate the positioning of Notch ligands Delta and Serrate (Ser) (Estella et al., 2012; Ruiz-Losada et al., 2018; Rauskolb, 2001). Notch activation in rings at the distal end of each segment not only directs the formation of the epithelial folds that prefigure the adult joints, but also contributes non-autonomously to appendage growth (Rauskolb and Irvine, 1999; Rauskolb, 2001; de Celis et al., 1998; Cordoba and Estella, 2020). At the tarsal segments, the Notch target gene *dysfusion* (*dysf*) controls epithelial folding and adult joint formation by spatially regulating Rho1 activity, a key regulator of the acto-myosin cytoskeleton, to drive apical cell constriction (Cordoba and Estella, 2018, 2014). In addition, apico-basal forces generated by patterned apoptotic cells may also contribute to epithelial folding (Monier and Suzanne, 2015; Manjon et al., 2007). These studies describe the molecular and cellular mechanisms implicated in the formation of epithelial folds at the local level, that is at the cells that invaginate. However, the impact of tissue-wide forces, such as those generated by cell proliferation, on the shape of these folds remains largely unexplored.

Here, we investigate the contribution of cell division to epithelial morphogenesis, using the leg disc tarsal folds as a model. We reveal that the interplay between cell proliferation, which generates tissue-wide compressive forces, and positional cues provided by Dysf govern the formation of epithelial folds. We show that Dysf specifies the location of the tarsal folds, providing the cells with unique mechanical properties that lead to the apical accumulation of F-actin and the apico-basal shortening of invaginating cells. Additionally, cell proliferation generates compressive stresses, contributing to the folding of the epithelium through the apico-basal elongation of interjoint cells, while simultaneously promoting the apical constriction of invaginating cells. In addition, we used a simple computer-based simulation model that recreated the initial process of tarsal fold formation. This model accurately predicts the folding phenotypes and the behavior of cells observed in the wild type and when cell proliferation is reduced or Dysf is absent.

## RESULTS

### Dynamics of leg epithelial fold formation

**Fig. 1.**
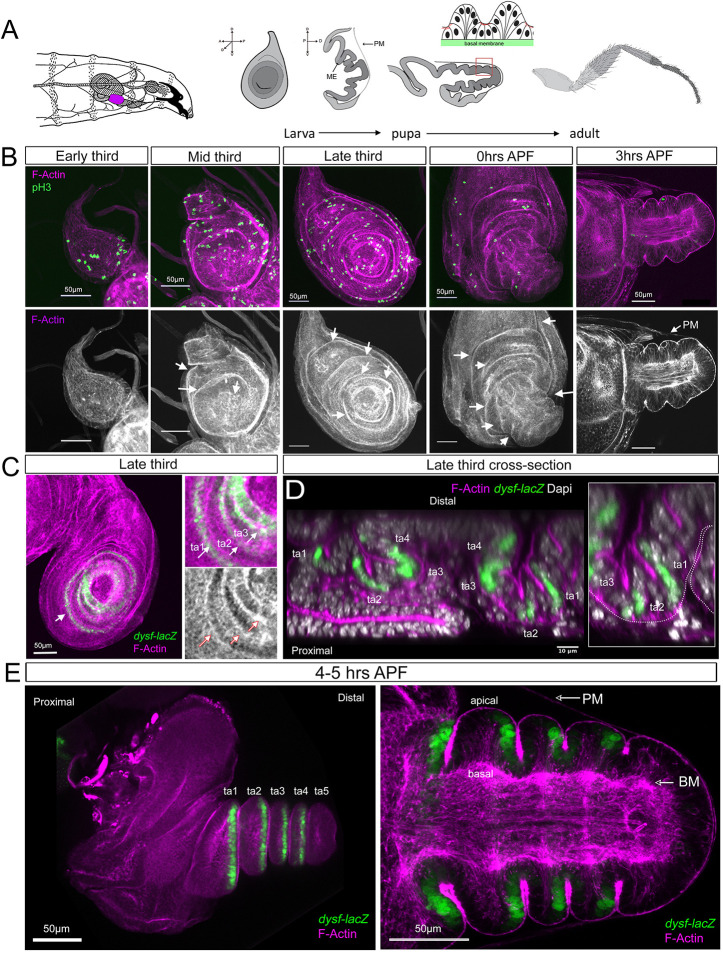
**Dynamics of cell proliferation and fold formation in the leg.** (A) Illustration of a third-stage larva with the imaginal leg disc colored in magenta and the development of the disc up to the adult stage. ME, main epithelium; PM, peripodial membrane. A detailed view of a prepupal tarsal fold is shown. Prepupal stage is considered as 0-12 h APF. (B) Time course of the leg imaginal disc at different time points of development stained for F-actin and pH3. Arrows indicate epithelial folds by F-actin accumulation. (C) Third instar leg imaginal discs stained for F-actin and *dysf-lacZ*. In the right panels, the formation of some tarsal folds (ta1-ta3) is indicated by arrows. (D) Cross-section of a third instar leg imaginal discs stained for F-actin, *dysf-lacZ* and Dapi. The four tarsal folds are indicated. A close up of the first and second tarsal joint is shown, with the basal side of the cells marked by a dotted line. (E) A 4-5 h APF leg imaginal disc stained for F-actin and *dysf-lacZ*. In the right panel a close-up view of the tarsal segments is shown in a sagittal section.

The leg disc comprises two distinct layers: a monolayer of pseudostratified epithelial columnar cells that gives rise to the adult appendage, referred to as the main epithelium (ME), and a layer of squamous cells called the peripodial membrane (PM) that confines the ME. In addition, an extracellular matrix (ECM) provides an elastic constraining environment that contributes to shape the appendages (Diaz-de-la-Loza et al., 2018; Pastor-Pareja and Xu, 2011) (Fig. 1A). In the third instar stage leg disc, the ME is characterized by concentric folding along the proximo-distal (PD) axis, which prefigures the division of the adult leg into segments that are separated by flexible joints (Rauskolb, 2001; de Celis et al., 1998). To explore the relationship between cell proliferation and epithelial folding, we used Phalloidin staining to visualize F-actin distribution, as well as phospho-histone 3 (pH3) and 5-ethynyl-2'-deoxyuridine (EdU) labeling to identify actively dividing cells. We also assessed the activity of a reporter for the tarsal Notch target gene *dysf* (Cordoba and Estella, 2014)*.* From early-third instar stage to 5 h after puparium formation (APF) (∼48 h), we observed the accumulation of F-actin in ring-like structures at folding points of the epithelium – these structures followed the sequential activation of the Notch pathway during leg disc development (Fig. 1B-D) (Rauskolb, 2001; de Celis et al., 1998; Condic et al., 1991). Dividing cells are found throughout the leg imaginal discs with no particular pattern and their number progressively decreases during early prepupal stages (Fig. 1B; Fig. S1). The distal folds that prefigure the adult tarsal joints initiate at mid-third instar and are fully recognizable at later stages distal to *dysf* expression (Fig. 1C-E).

### Cell proliferation is required for leg epithelial folding and adult joint formation

**Fig. 2.**
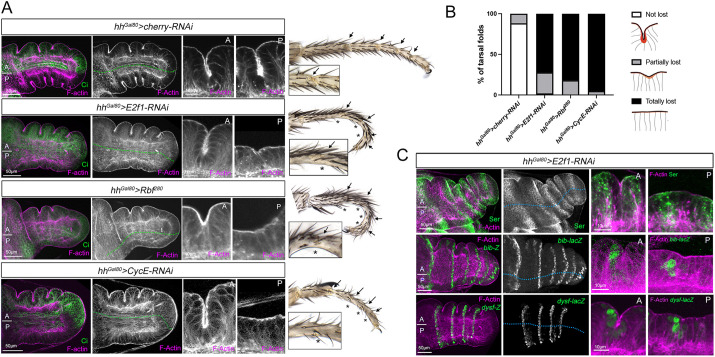
**Reduction of cell proliferation inhibits epithelial folding and joint formation.** (A) Tarsal region of prepupal leg imaginal discs expressing the indicated transgenes for 48 h by *hh^Gal80^>* stained for F-actin, and Cubitus interruptus (Ci) that marks the anterior compartment. The *cherry-RNAi* line was used as control. The anterior (A) and posterior (P) compartments and their boundary are represented by a dotted green line. A higher magnification of an anterior and posterior sagittal view of tarsal folds is presented for each genotype. The respective adult leg phenotypes of these experiments are shown, with the tarsal joints indicated by arrows, or by asterisks if affected. A representative close view of a tarsal joint is also presented. (B) Quantification of tarsal fold defects for the prepupal legs shown in A. The genotypes are as follows: control *hh^Gal80^>cherry-RNAi* (24 legs, 91 folds), *hh^Gal80^>E2f1-RNAi* (37 legs, 101 folds), *hh^Gal80^>Rbf^280^* (21 legs, 61 folds), *hh^Gal80^>CycE-RNAi* (7 legs, 21 folds). The larvae from these genotypes were kept at 17°C and transferred to 31°C at the beginning of third instar. C) Prepupal leg discs of the *hh^Gal80^>E2f1-RNAi* genotype dissected 48 h after inducing the transgene in the posterior compartment and being stained for F-actin, Ser, *bib-lacZ* and *dysf-lacZ*. Engrailed and Ci staining was used to demarcate the posterior and anterior compartments, respectively. The antero-posterior compartment boundary is represented by a dotted blue line. A higher magnification of an anterior and posterior tarsal fold is indicated for each staining.

As leg epithelial folding is taking place in a highly proliferative tissue, we investigated the influence of cell division on tarsal morphogenesis. We employed a combination of the *hedgehog* (*hh*)*-Gal4* driver, the *tub-Gal80^ts^* and *UAS-E2f1* RNAi constructs (*hh^Gal80^>E2f1-RNAi*) to temporally knockdown E2f1, a key regulator of the cell cycle (Ohtani et al., 1995), in the posterior compartment for 48 h (Fig. 2A; Fig. S2A,B). We found that this treatment reduced the number of cells in the posterior distal leg domain by 35% compared with a posterior control compartment (Fig. S2C,D). E2f1 knockdown has a strong effect on the formation of the prepupal tarsal epithelial folds (Fig. 2A,B): in the control anterior compartment the characteristic tarsal folds are observed, whereas in the posterior compartment the epithelium is flattened and the adult tarsal joints are lost (Fig. 2A,B). To confirm our findings, we also suppressed E2f1 function by expressing an activated form of the E2f1 repressor Retinoblastoma (Rbf^280^) (Xin et al., 2002) and by knocking down CycE (Ohtani et al., 1995). Consistent with our previous observations, both methods produced comparable results (Fig. 2A,B).

This absence of leg epithelial folding may be attributed to a failure in joint specification. To test this possibility, we stained for the Notch ligand Ser and for two Notch target genes: *big brain* (*bib*) and *dysf* (de Celis et al., 1998; Cordoba and Estella, 2014; Rauskolb, 2001). Although the knockdown of E2f1 in the posterior compartment prevented epithelial folding, no defects in the expression of *Ser*, *bib* or *dysf* were observed (Fig. 2C), which suggests that the absence of leg folding is not a direct consequence of the lack of Notch activity.

To extend these results we used the *apterous* (*ap*)-*Gal4* to reduce cell division in a restricted domain of the leg, the fourth tarsal segment encompassing the ta4/ta5 fold. The knockdown of E2f1 or CycE, or the expression of *Rbf^280^*, in this domain has a strong effect on the formation of the adult ta4/ta5 fold and joint without affecting *dysf-lacZ* expression (Fig. S3). To determine whether cell division is specifically necessary in folding cells or at the tissue level, we knocked down E2f1 using *dysf-Gal4* and *bib-Gal4*. Both lines are activated in the future tarsal folds, starting at third instar stage (Cordoba and Estella, 2014; Manjon et al., 2007). Tarsal epithelial folds and adult joints were still formed under these experimental conditions (Fig. S4).

Our results suggest that cell proliferation is required for epithelial folding and adult tarsal joint formation. Importantly, this requirement is not specifically needed at the folding cells.

### Rho1 activity and apical constriction at the tarsal epithelial folds are impaired in proliferation-deficient epithelia

**Fig. 3.**
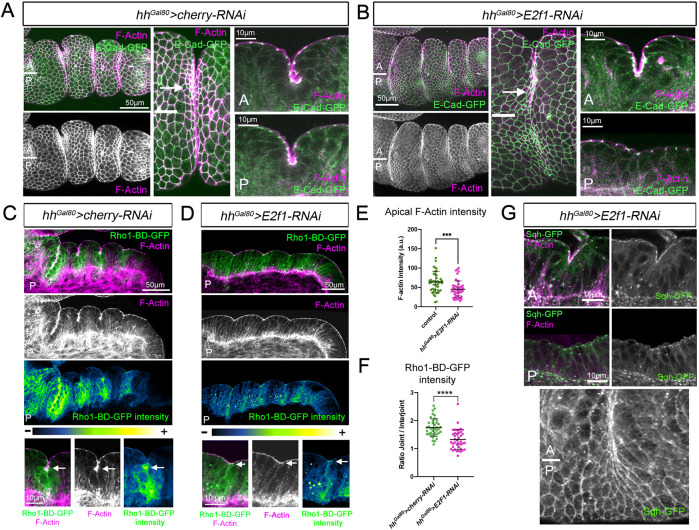
**Analysis of F-actin, Rho1 activity and Sqh after E2f1 knockdown.** (A,B) Tarsal region of prepupal leg imaginal discs expressing for 48 h the indicated transgenes by *hh^Gal80^>* stained for F-actin and E-Cad. The anterior (A) and posterior (P) compartment boundary is represented by a white line. A higher magnification of an anterior and posterior sagittal view of tarsal folds is presented for each genotype. Arrows indicate F-actin accumulation in control anterior compartment cells that are apically constricted. (C,D) Posterior tarsal region of prepupal leg imaginal discs expressing *Rho1-BD-GFP* and the indicated transgenes by *hh^Gal80^>* for 48 h and stained for F-actin. Regions of enhanced GFP levels (GFP and false color) are seen at joints in C that are separated by regions of lower GFP levels in interjoint regions. This pattern is lost in *hh^Gal80^>E2f1-RNAi*, where GFP accumulation in the folding region is reduced. Higher magnification views of a sagittal section of a posterior tarsal fold show a separate channel for F-actin and false color to enhance contrast. GFP and F-actin levels are accumulated apically in C in the cells that are undergoing apical constriction (arrow), whereas in D, less accumulation of GFP and F-actin is observed. (E) Quantification of apical F-actin (mean intensity) at the joint cells in the anterior (control) and posterior (experimental) compartment of *hh^Gal80^>E2f1-RNAi* prepupal legs. A total of 16 legs and 46 joints were quantified per genotype. (F) Ratio of fluorescence levels (mean intensity) of Rho1-BD-GFP within joint and interjoint domains in *hh^Gal80^>cherry-RNAi* (control, 13 legs, 40 joints and interjoints) and *hh^Gal80^>E2f1-RNAi* (14 legs, 41 joints and interjoints) prepupal legs. A ratio of 1 implies the same levels in joint and interjoint domains, whereas any increment over 1 means higher levels in the joint versus interjoint domain. (G) Higher magnification of anterior and posterior sagittal views of tarsal folds from *hh^Gal80^>E2f1-RNAi* prepupal legs stained for F-actin and Sqh-GFP. An apical view of a tarsal joint where the anterior and posterior compartments are indicated showing the accumulation of Sqh-GFP in the control anterior compartment cells that are apically constricted. ***P<0.0005, ****P<0.0001 (unpaired two-tailed Student's *t*-test). Error bars represent s.d.

Localized Rho1 activation by Dysf promotes epithelial folding through cell shape changes such as apical constriction (Cordoba and Estella, 2018). Apical constriction is mediated by the shrinkage of the apical cortex surface by the acto-myosin networks and forces are transmitted to the neighboring cells through the adherens junction attachments (Martin and Goldstein, 2014). To investigate the impact of cell proliferation on the cell shape changes necessary for epithelial folding, we downregulated E2f1 in the posterior compartment and examined apical constriction by staining for E-Cadherin and F-actin. We observed that cells at the presumptive joint fold in the posterior compartment failed to properly constrict apically and showed significantly less apical F-actin than control anterior cells (Fig. 3A,B,E). Next, we monitored Rho1 activity using a GFP-based sensor (Rho1-BD-GFP) which contains the Rho1 binding domains of Protein kinase N fused to GFP and that recognizes the active form of Rho1 (Simoes et al., 2006). Expression of the *UAS-Rho1-BD-GFP* construct under the *hh-Gal4* driver showed a strong accumulation of GFP in the tarsal folds and specifically in the apical domain of the cells that constrict apically (Fig. 3C) (Cordoba and Estella, 2018). Importantly, GFP accumulation, and therefore Rho1 activity, is reduced but not eliminated in the cells of the fold region where cell proliferation was decreased (Fig. 3D,F). Next, we followed the localization of the Myosin II regulatory light chain using a GFP-tagged version of Spaghetti squash (Sqh-GFP). Sqh is located subapically at the adherens junctions and, although its apical distribution does not change after E2f1 knockdown, we observed less accumulation than in control anterior cells (Fig. 3G).

These results suggest that cell proliferation, together with Dysf, contributes to Rho1 activation, promoting the acto-myosin network contraction and apical constriction of cells forming the presumptive joint fold.

### Tissue compression generated by cell proliferation is necessary for leg epithelial folding

**Fig. 4.**
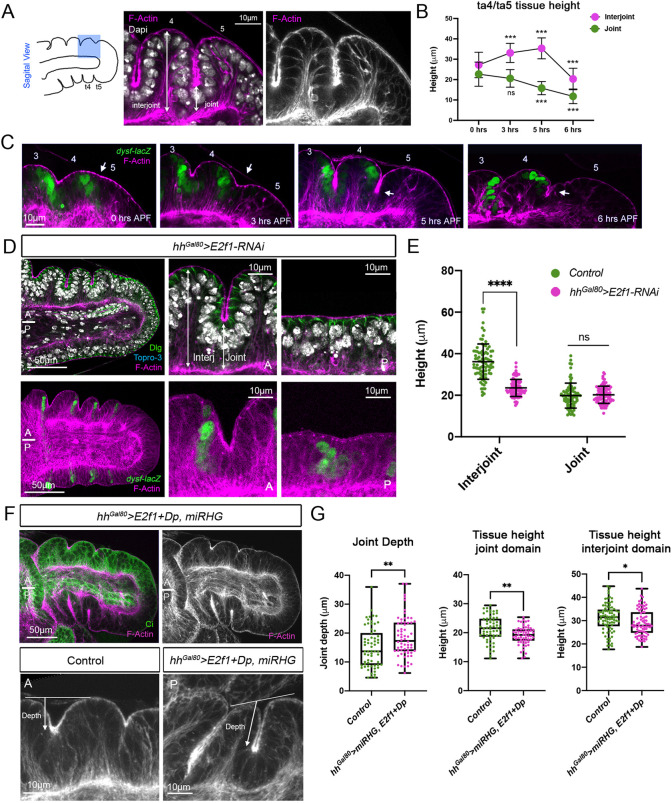
**Cell proliferation promotes epithelial folding at specific locations.** (A) Schematic depicts a sagittal view of the tarsal domain in a prepupal leg, highlighting the ta4/ta5 tarsal fold region in blue. Also shown is the ta4/ta5 tarsal fold stained with Dapi and F-actin. The interjoint and joint domains are indicated. (B) Quantification of tissue height of the ta4/ta5 interjoint and joint domains from 0 h to 6 h APF (0 h, 34 joints, 34 interjoints; 3 h, 24 joints, 24 interjoints; 5 h, 26 joints, 26 interjoints; 6 h, 30 joints, 30 interjoints). Error bars represent s.d. ***P<0.001 [one-way ANOVA, comparing each time point with the control (0 h) as indicated]. ns, not significant. (C) Time course imaging the ta4/ta5 joint (arrow) at different time points stained for F-actin and *dysf-lacZ*. (D) *hh^Gal80^>E2f1-RNAi* prepupal leg discs dissected 48 h after inducing the transgene in the posterior compartment and stained for F-actin, Dlg and Topro-3, or *dysf-lacZ*. The antero-posterior compartment boundary is represented by a white line. A higher magnification of an anterior (A) and posterior (P) tarsal fold is indicated for each staining. (E) Quantification of tissue height at interjoint and joint domains in anterior (control) and posterior (experimental) compartment of *hh^Gal80^>E2f1-RNAi* (21 legs, 62 joints, 78 interjoints) prepupal legs. ****P<0.0001 (unpaired two-tailed Student's *t*-test). ns, not significant. Error bars represent s.d. (F) Prepupal leg imaginal discs of the *hh^Gal80^>E2f1+Dp, miRHG* genotype dissected 24-30 h after inducing the transgene in the posterior compartment and stained for F-actin and Ci. The antero-posterior compartment boundary is represented by a white line. A higher magnification of an anterior and posterior tarsal fold is indicated. The anterior compartment is used as a control. Also indicated is how the joint depth is measured. (G) Quantification of joint depth and tissue height at the interjoint and joint domains of the prepupal legs in F. A total of 22 legs were dissected and 65 joints and 87 interjoints were measured. *P<0.05, **P<0.01 (unpaired two-tailed Student's *t*-test). Box plots show median values (middle bars) and first to third interquartile ranges (boxes); whiskers indicate the minimum and maximum points. Dots are individual data points.

Our results suggest that compressive stresses generated during cell division in the imaginal disc could contribute to epithelium folding. To gain a more comprehensive understanding of the folding mechanism, we measured the height of the interjoint and joint domains before and after the formation of the ta4/ta5 tarsal fold using live imaging and fixed tissue (see Materials and Methods). Although this particular fold is the last to form, between 0 and 6 h after puparium formation (APF), it also requires cell division to properly fold (Fig. S3). While the height of the joint fold (distal to *dysf*) gradually decreases, the height of the interjoint domain (proximal to *dysf*) progressively increases until ∼5 h APF, when it suddenly drops (Fig. 4A-C; Fig. S5; Movie 1). At this time point (6 h APF), basal ECM degradation and the removal of the peripodial membrane allow for leg expansion (Diaz-de-la-Loza et al., 2018; Proag et al., 2019). The increase in cell height in the interjoint domain is appreciated by the long actin filaments that attach the cells to the basement membrane (Fig. 4A,C).

Next, we analyzed interjoint and joint domain height after reducing cell proliferation in the posterior compartment, and compared it with the anterior control. We used *dysf-lacZ* as a marker for the region where the joints will form. We found that E2f1 knockdown led to a significant reduction in the interjoint height that resulted in the suppression of the upward fold (Fig. 4D,E). In contrast, the relative height of the joint domain does not significantly change when compared with the anterior control cells (Fig. 4D,E). These results suggest that compressive forces generated by cell proliferation promote the upward elongation of the interjoint epithelium and thus contribute to the formation of the tarsal folds.

Next, we analyzed the effects of an increase in cell proliferation on tissue folding. To this end, we ectopically expressed E2f1, and its obligated partner Dp, in the posterior compartment. As E2f1 not only promotes cell proliferation but also cell death, we inhibited apoptosis using the *UAS-miRHG* construct that represses the activity of the proapoptotic genes (Siegrist et al., 2010; Moon et al., 2008). We also stimulated proliferation by the induction of the transcriptional effector of the Hippo pathway, Yorkie (Yki) (Huang et al., 2005). We employed the *hh^Gal80^* system to restrict the expression of these transgenes to 24-30 h before pupal dissection. This was done to avoid deformation of the leg epithelium, as we have observed that prolonged expression times can prevent an accurate phenotypic analysis. In both experiments, although the overall morphology and location of the leg epithelial folds were maintained, we detected an increase in the depth of the invaginated joint region that was accompanied by a decrease in the height of this domain when compared with the control compartment (Fig. 4F,G; Fig. S6). However, we did not observe an expansion on the height of the interjoint domain, suggesting that probably these cells have reached the maximum of their elongation.

These observations suggest that an increase in tissue compression due to cell proliferation promotes epithelial folding at specific positions. In the case of the leg discs, these positions at the tarsal segments are dictated by Dysf.

### The extracellular matrix contributes to generate the compressive forces that promote the folding of the epithelium

Imaginal discs grow surrounded by the ECM, which acts as an elastic constraining element, shaping them during morphogenesis (Pastor-Pareja and Xu, 2011; Diaz-de-la-Loza et al., 2018; Harmansa et al., 2023). To investigate the role of the ECM in promoting leg folding and joint formation, we first analyzed Collagen-IV, a major component of the basal matrix, using a GFP-tagged version of the Viking protein (Vkg-GFP).

**Fig. 5.**
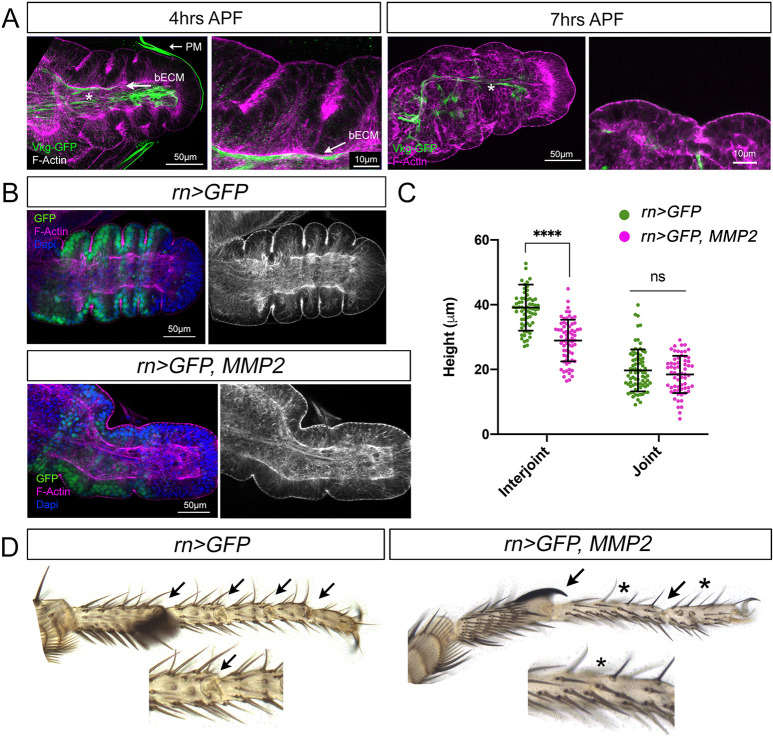
**The extracellular matrix generates compressive forces that promote epithelium folding.** (A) Tarsal region of prepupal leg imaginal discs dissected 4 h APF and 7 h APF and stained for F-actin and Vkg-GFP. Vkg-GFP covers the basal surface of the main epithelium and of the peripodial membrane at 4 h APF. Note Vkg-GFP degradation in epithelial cells and peripodial membrane at 7 h APF. Asterisk shows Vkg-GFP accumulation at the tendon precursors and other internal structures. A detailed image of a tarsal joint is shown. Arrows indicate the basal extracellular matrix (bECM) and the peripodial membrane (PM). (B) Defective fold formation after *MMP2* expression in the tarsal segments (*rn-Gal4*, *UAS-GFP*; *UAS-MMP2*) of 3-4 h prepupae leg discs stained for F-actin, GFP and Dapi. Note that the leg is expanded compared with its control (*rn-Gal4, UAS-GFP)*. (C) Quantification of tissue height at the interjoint and joint domains in *rn>GFP* (control, 12 legs, 77 joints, 65 interjoints) and *rn>GFP, MMP2* (12 legs, 63 joints, 66 interjoints) prepupal legs dissected between 3-4 h APF and stained for Dysf and F-actin. ****P<0.0001 (unpaired two-tailed Student's *t*-test). ns, not significant. Error bars represent s.d. (D) Tarsal segments from adult legs expressing the indicated transgenes as in B. Tarsal joints are indicated by arrows and the affected ones by asterisks. A representative close view of a tarsal joint is shown.

As previously reported (Diaz-de-la-Loza et al., 2018), we observed that the main epithelial cells are attached to the basal ECM at ∼4 h APF (Fig. 5A). At later stages of pupal development (∼7 h APF), ECM remodeling and removal of the peripodial membrane initiate leg expansion along the proximo-distal axis (Fig. 5A) (Milner et al., 1984; Proag et al., 2019; Diaz-de-la-Loza et al., 2018; Pastor-Pareja and Xu, 2011).

Next, we tested whether removal of the basal ECM affects fold and adult joint formation. To this end we overexpressed Matrix metalloproteinase 2 (MMP2) in the distal domain of the leg with the *rotund* (*rn*) *Gal4* (Fig. 5B). We found that the epithelial folds are severely disrupted as the distal appendage is prematurely expanded (Fig. 5B). Quantification of tissue height revealed the apico-basal shortening of interjoint cells in this experimental condition (Fig. 5C). Remarkably, basal ECM degradation also affects the formation of some adult joints (Fig. 5D). These results confirm the role of the ECM in promoting epithelial folding.

### Dysf induces apical F-actin accumulation and cell shortening

Previous work from our lab has elucidated the role of the Notch target Dysf in tarsal folds and adult joint formation (Cordoba and Estella, 2014, 2018, 2020). Dysf enhances Rho1 activity and F-actin accumulation, and promotes apical constriction at the presumptive joint cells (Cordoba and Estella, 2018). This suggests that Dysf confers a unique property to these cells by preventing their apico-basal elongation following tissue compression.

**Fig. 6.**
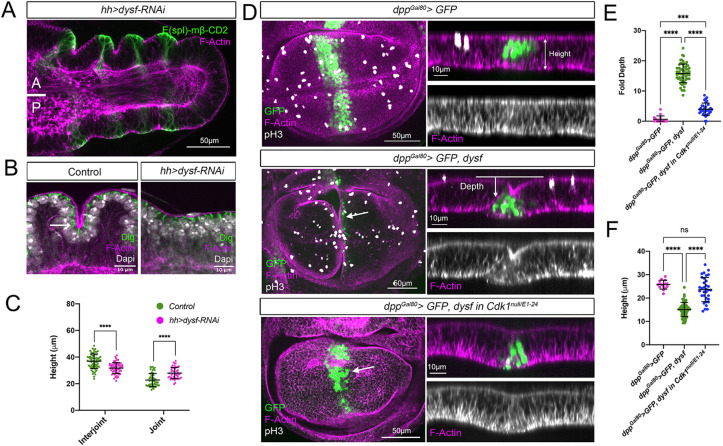
**Dysf induces F-actin accumulation and cell shortening.** (A) Tarsal region of prepupal leg imaginal discs expressing *UAS-dysf-RNAi* in the posterior compartment by *hh-Gal4* (*hh>*) stained for F-actin and for the Notch target gene *E(spl)mβ-HLH*. Anterior (A) and posterior (P) compartments are indicated and their boundary is represented by a white line. (B) Higher magnification of anterior (control) and posterior sagittal views of tarsal folds from a prepupal leg disc expressing the *UAS-dysf-RNAi* in the posterior compartment and stained for F-actin, Dlg and Dapi. Arrow indicates F-actin accumulation in the control anterior compartment cells that are apically constricted. (C) Quantification of tissue height at the interjoint and joint domains in anterior (control) and posterior (experimental) compartment of *hh>dysf-RNAi* (17 legs, 51 joints, 68 interjoints) prepupal legs. ****P<0.0001 (unpaired two-tailed Student's *t*-test). Error bars represent s.d. (D) Apical view and *z*-section of the pouch region of wing imaginal discs expressing the indicated transgenes for 24 h under the *dpp^Gal80^>* line in a wild-type or *Cdk1^null/E1-24^* background (see Materials and Methods). The *dpp-Gal4* driver was used to express *GFP* and *dysf* in a band of cells of the anterior compartment of the wing pouch. Discs stained for F-actin, pH3 and GFP. The fold generated by the ectopic expression of *dysf* is marked by an arrow. Also indicated is how the fold depth and tissue height are measured. (E,F) Quantification of fold depth (E) and tissue height (F) in the GFP domain measured in the *z*-sections of the genotypes described in D. *dpp^Gal80^>GFP* (*n*=15), *dpp^Gal80^>GFP, dysf* (*n*=32) and *dpp^Gal80^>GFP, dysf* in *Cdk1^null/E1-24^* (*n*=18). Two measurements were taken for each disc in the experimental conditions at different locations. ***P<0.001, ****P<0.0001 (one-way ANOVA). ns, not significant. Error bars represent s.d.

To explore this possibility, we analyzed the behavior of the interjoint and joint domain cells following Dysf depletion in the posterior compartment (*hh>GFP*, *dysf-RNAi*). As previously reported, the posterior domain loses the characteristic tarsal folds while maintaining Notch activation, as visualized by the Notch target *Enhancer-of-split mβ, helix-loop-helix* [*E(spl)mβ-HLH*] (Fig. 6A) (Cordoba and Estella, 2014, 2018; de Celis et al., 1998). Interestingly, we found that the apico-basal shortening of the corresponding joint domain does not occur in the absence of Dysf (Fig. 6B,C). We also noticed that the height of the interjoint domain decreased compared with the control anterior domain (Fig. 6B,C). Importantly, cell proliferation was not affected in the absence of Dysf (Fig. S7).

To confirm that Dysf promotes the apico-basal shortening of the cells, we ectopically expressed *dysf* in a stripe of cells within the relatively flat epithelium of the wing pouch using the *dpp-Gal4*, *tub-Gal80^ts^* line (*dpp^Gal80^>*) (Fig. 6D). Dysf is sufficient to increase the accumulation of F-actin, induce the formation of an ectopic fold and reduce the apico-basal height of the domain (Cordoba and Estella, 2018) (Fig. 6D). Importantly, fold induction by Dysf depends on cell proliferation, as temporarily blocking cell division throughout the disc using a temperature-sensitive allele of *Cdk1* (see Materials and Methods) prevents the accumulation of F-actin and the formation of ectopic folds (Fig. 6D-F). However, blocking cell proliferation specifically in cells expressing *dysf* (*dpp^Gal80^>dysf*, *Cdk1-RNAi* or *E2f1-RNAi*) has no or minimal effect on its ability to induce a fold (Fig. S8). These results suggest that cell proliferation is required at a tissue-wide level for the induction of the folds, as we have described in the leg.

### Cell proliferation is required for epithelial folding in wing imaginal discs

**Fig. 7.**
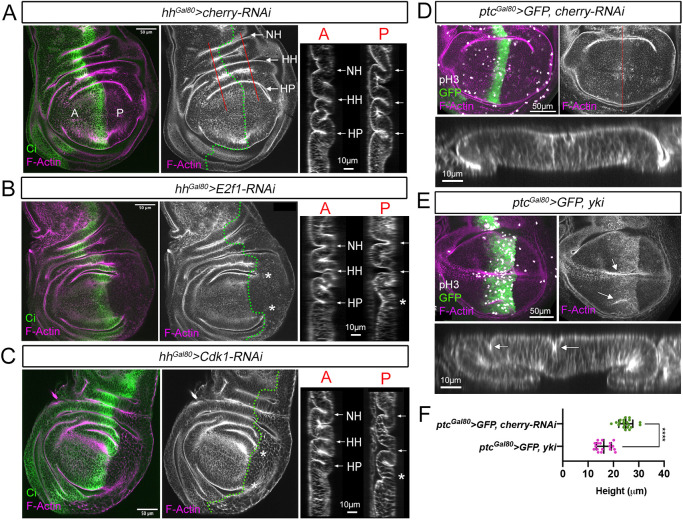
**Cell proliferation is required for the correct formation of the hinge-pouch fold.** (A-C) Third instar wing imaginal discs stained for F-actin and Ci expressing the indicated transgenes for 48 h in the posterior compartment with the *hh^Gal80^>* driver. The antero-posterior compartment is indicated by a green dotted line and the regions selected for the *z*-sections in the anterior (A) and posterior (P) compartment with a red line. The different folds in the wing disc are indicated with arrows: HH, hinge-hinge; HP, hinge-pouch; NH, notum-hinge. Asterisks indicate the absence or incomplete formation of a fold. (D,E) Apical view and a representative *z*-section of *ptc^Gal80^>* wing imaginal discs expressing *UAS*-*cherry-RNAi* (D) or *UAS*-*yki* (E) for 48 h. The imaginal discs were stained for F-actin, pH3 and GFP. A *z*-section of the pouch region is indicated by a red line and shown below. The ectopic folds are indicated by arrows. (F) Quantification of tissue height in the GFP domain measured in the *z*-sections of the genotypes described in E and F, D and E. *ptc^Gal80^>GFP, cherry-RNAi* (*n*=20) and *ptc^Gal80^>GFP, yki* (*n*=18)*.* ****P<0.0001 (unpaired two-tailed Student's *t*-test). Error bars represent s.d.

As in the leg, the wing imaginal disc is folded in a stereotyped manner. It has been proposed that planar differential rates of cell division initiate the folding of the wing epithelium (Tozluoglu et al., 2019). However, other reports have questioned the relevance of cell proliferation for wing fold formation (Sui et al., 2018). Our results in the leg disc demonstrated that cell division is required for epithelial folding. Therefore, we tested the consequences of altering cell proliferation for the correct folding of the wing epithelium. First, we knocked down E2f1 or Cdk1 in the posterior compartment during third instar stage and studied the formation of the three main epithelial folds: notum-hinge (NH), hinge-hinge (HH) and hinge-pouch (HP). These folds are readily observed in mature wing imaginal discs (Fig. 7A). As observed in Fig. 7B,C, reducing cell proliferation inhibits the correct formation of the HP fold, but not the other two folds (NH and HH). One possibility is that the NH and HH folds do not require cell proliferation to form. However, given that the HP fold is the last one to be formed (Tozluoglu et al., 2019; Sui et al., 2012), it is more likely that this fold is the only one that is affected at the time we reduced proliferation. We then examined whether an increase in cell proliferation was sufficient to induce epithelial folding. To test this, we expressed *yki* for 48 h before dissection in a stripe of cells in the wing disc pouch using the *patched-Gal4, tubGal80ts (ptc^Gal80^>)* driver (Fig. 7D-F). Notably, *yki* induced overproliferation, as visualized by an increase of pH3 mitotic cells, and the formation of ectopic folds (Fig. 7E,F). These results indicate that an increase of cell proliferation is sufficient to force the formation of epithelial folds; however, these appear at random positions within the *yki*-expressing cells.

### Simulation of leg epithelial folding

Our results in the leg discs indicate that cell proliferation, within the constraints of the ECM, generates tissue-wide compression forces that, in collaboration with the patterned activity of Dysf, promotes epithelial folding.

**Fig. 8.**
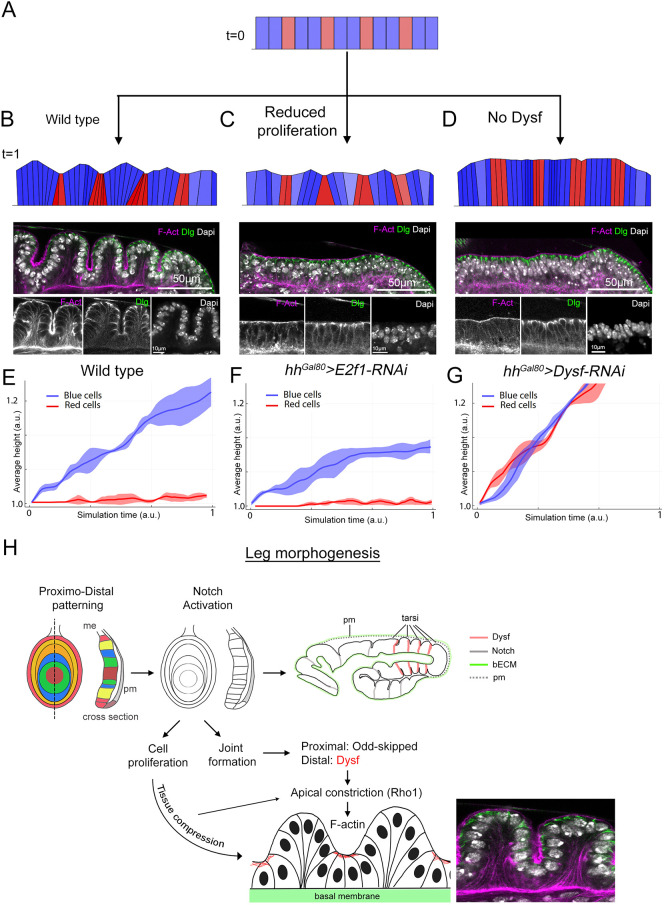
**Simulation of leg epithelial folding and proposed model.** (A) Initial stage (t=0) of the mathematical simulation of the early events of tarsal epithelial folding. The different colors indicate the interjoint (blue) and joint (red) domains. (B-D) Final stage of the simulation (t=1) after allowing the cells to proliferate normally (B), by reducing proliferation (C) and by removing the restriction to grow apically (D). See the text and Movies 2-4 for full details. Under each illustration, the corresponding *in vivo* experiment is presented as a sagittal view of the tarsal epithelium that results from knocking down E2f1 or Dysf in the posterior compartment stained for F-actin, Dlg and Dapi. E2f1 knockdown image is the same as Fig. 4D but flipped to maintain the orientation to the rest of the images. (E-G) Quantification of the average blue and red cell height for each experimental condition of the simulation. The line represents the average of three simulations and the ribbon the standard deviation. (H) Proposed model for leg epithelial folding. Leg subdivision in different domains of gene expression along the PD axis allows the sequential activation of the Notch pathway in concentric rings. Notch is required for the non-autonomous growth and for the formation of the epithelial folds that prefigure the adult joints. In the proximal domain, Notch activates the odd-skipped family of transcription factors, whereas in the tarsal region it induces *dysf* expression. The combined effect of tissue compression generated by the proliferation of leg imaginal cells, within the constraining environment of the ECM and the peripodial membrane, and Dysf promotes Rho1 activation and the apical accumulation of F-actin in a specific group of cells, leading to their apical constriction and inability to grow apically (joint cells). In contrast, interjoint cells respond to tissue compression by expanding apically, promoting the folding of the epithelium. bECM, basal extracellular matrix; me, main epithelium; pm, peripodial membrane.

To test whether the combination of cell proliferation and *dysf* local expression is sufficient to initiate the periodic folding profile observed during the early steps of tarsal joint formation, we developed a computer-based simulation model of a simple monolayer of cells set to follow certain basic rules: (1) all cells can grow and proliferate; (2) dimensions of the system in the *x*-axis are fixed, so the tissue is not able to freely expand in this direction – this restriction mimics the effect of the ECM and peripodial membrane; (3) each cell grows in volume by compressing its neighbors until they reach a minimal size; (4) when neighbors reach this minimal size, cells tend to grow by expanding apically and upwards; (5) specific locations in the tissue are defined in such a way that cells inside these domains (marked as red in Fig. 8) cannot expand apically, as occurs with the cells that are influenced by Dysf in the joint domain. To simplify the model, the red cells (joint) do not reduce their apico-basal length, in contrast to what happens during *in vivo* folding (Fig. 4B,C).

With these five simple assumptions, and starting from a limited number of cells confined in the horizontal direction (Fig. 8A), we ran computational simulations for different conditions. In wild-type situations (Fig. 8B,E; Movie 2) cells proliferate, increasing the density of the tissue until they reach a minimal size, at which point blue cells (interjoint domain cells) start to grow apically, compressing the apical domain of red cells (joint domain cells). As red cells cannot elongate apically, they expand by compressing the basal domain of their neighbors. The combination of these two processes results in the spontaneous formation of folds simulating the characteristic folding pattern of the distal leg (Fig. 8B; Movie 2).

Our experiments provide evidence that a reduction in proliferation or the knockdown of Dysf aborts leg epithelial folding (Figs 2A and 6A). To simulate these conditions, we first slowed down cell cycle duration to match the number of cells observed in the E2f1 knockdown experiments (35% reduction). In these conditions, tissue folding is impaired as cells never reach the confluence required for compressing their neighbors to the extent that they need to expand apically (Fig. 8C,F; Movie 3). In this simulation, we observed that joint domain cells (red) suffer less compression, which is translated in less apical constriction (Fig. S9). A similar situation is observed in our experiments after E2f1 knockdown (Fig. 3B).

Secondly, we modeled the absence of Dysf by performing simulations where the restriction on apical elongation in red cells was eliminated, allowing all cells to expand apically if necessary, mirroring the observations in our experiments (Fig. 6A-C). In these conditions, tissue folding is also impaired as no differential apical growth is established between joint and interjoint cells (Fig. 8D,G; Movie 4). In addition, we detected the absence of apical constriction in any location of the tissue, as observed experimentally (Fig. S9) (Cordoba and Estella, 2018).

## DISCUSSION

In this study, we investigated how cell proliferation, through the accumulation of cell mass, serves as a tissue-wide mechanism leading to epithelium folding. During larva development, the leg imaginal disc grows restricted by the ECM and the peripodial membrane. These elements, together with the accumulation of cells, help generate the compressive forces that fold the epithelium at specific positions. In the tarsal segments these positions are defined by the Notch target gene *dysf*. Here, we found that cell proliferation not only contributes to shaping the leg epithelium but is also necessary for the formation of the adult joints.

Previous work has detailed the mechanisms that promote the cell shape changes required for epithelial folding (reviewed by Tozluoglu and Mao, 2020). However, the influence that tissue-wide mechanisms have on epithelial folding has been less explored. In the *Drosophila* embryo, a genetic program directs the apical constriction of a group of cells through changes in the acto-myosin network to form the ventral furrow (reviewed by Gheisari et al., 2020). Importantly, these cell shape changes are coordinated with embryonic-wide ectodermal movements towards the furrow that facilitate internalization of mesodermal cells (Rauzi et al., 2015; Guo et al., 2022). In the wing disc, the position and formation of the epithelial folds is controlled by a patterning genetic network (Sui and Dahmann, 2020b; Sui et al., 2012; Villa-Cuesta et al., 2007; Wang et al., 2016), which is translated into mechanical forces that change the shape of the cells that form the folds (Sui et al., 2018; Sui and Dahmann, 2020a). In addition, differential planar growth rates in the disc combined with the constraining effects of the basement membrane helps the epithelium to fold at precise positions (Tozluoglu et al., 2019). All these studies highlight the importance of tissue-wide forces to act coordinately with local mechanisms to promote tissue folding and organ shape.

In the developing leg imaginal disc, local activation of Notch at the distal end of each segment not only induces epithelial folding but also regulates appendage growth in a non-autonomous manner (Fig. 8H) (Cordoba and Estella, 2020; Ruiz-Losada et al., 2018; Rauskolb, 2001; de Celis et al., 1998; Rauskolb and Irvine, 1999). In the tarsal segments, the Notch target Dysf transcriptionally controls the patterned activation of Rho1 to drive apical constriction and epithelial folding (Cordoba and Estella, 2014, 2018).

Leg epithelial folds appear sequentially from the beginning of the third instar larval stage, a time of active cell proliferation. Our experiments, which involved reducing cell division rates during the folding process, indicate that a minimal number of cells are required to generate the characteristic folds in the tarsal epithelium. Importantly, the failure of the epithelium to fold is not due to defects in Notch activation, as the expression of its target genes, *dysf* and *bib*, is not affected. Instead, we observed that Rho1 activation and F-actin accumulation in joint domain cells, processes that depend on Dysf activity (Cordoba and Estella, 2018), are impaired after reducing cell proliferation rates. These results suggest that tissue compression reinforces Rho1 activation and apical constriction induced by Dysf. Nevertheless, the exact mechanism responsible for this effect remains unknown.

A detailed analysis of the phenotypes resulting from reduced cell numbers uncovers a previously unanticipated behavior of the interjoint cells: their ability to increase in height in response to tissue compression. These data suggest that at least two processes operate in parallel for the proper formation of tarsal folds (Fig. 8H). The first one is the activation of *dysf* at the distal end of each tarsal segment. Dysf promotes Rho1 activation and the apical constriction of the cells that is accompanied by a decrease in their height (Cordoba and Estella, 2018). At the same time, cell proliferation within the constraining environment of the ECM and the peripodial membrane generates tissue-wide compressive forces that promote different cell behaviors depending on the developmental fate of the cells. The progressive accumulation of cells forces the apico-basal stretch of the interjoint cells, whereas it facilitates apical constriction through the activation of Rho1 and the apical accumulation of F-actin at the joint domain cells. Moreover, compressive forces generated by apical constricting cells at the joint domain also favor the apico-basal elongation of interjoint cells and the formation of the folds.

Aside from cell proliferation, additional mechanisms likely contribute to tissue compression and facilitate epithelial fold formation, including oriented cell division, cell intercalation and cell shape changes (Taylor and Adler, 2008; Baena-Lopez et al., 2005; Condic et al., 1991). Employing live imaging to track cell behavior and dynamics during fold formation, although challenging, promises to provide valuable insights.

Using the wing disc, we were also able to demonstrate that cell proliferation is sufficient to induce epithelial folding and is, at least, required for the formation of the HP fold. Interestingly, non-uniform growth rates contribute to the correct location of the folds at the time of their formation, confirming that cell proliferation and the genetic patterning mechanisms act coordinately to shape tissues (Tozluoglu et al., 2019).

In this work we also recreated the initial steps of tarsal fold formation using a simple computer-based simulation model. Here, all cells proliferate in a confined space, as happens *in vivo* because of the presence of the ECM and the peripodial membrane, and the patterned Dysf function is generated by preventing cells to grow apically due to F-actin accumulation. In this model, cells compress their neighbors until they reach a minimum volume, ultimately resulting in the formation of folds. As occurs *in vivo*, the interjoint cells increase in height while joint cells progressively constrict their apical side and expand basally generating the folding observed in the tarsal segments. Remarkably, this simple model predicts the folding phenotypes and the behavior of the cells after reducing cell proliferation and in the absence of Dysf.

In summary, this work emphasizes the importance of studying both local and tissue-wide mechanisms that together orchestrate the formation of epithelial folds and organ architecture in a reproducible pattern.

## MATERIALS AND METHODS

### *Drosophila melanogaster* lines

The culture of *Drosophila melanogaster* strains was carried out in 12 h light/12 h dark cycles in incubation chambers under controlled temperature (17°C, 25°C or 31°C, depending on the experiments). The following Gal4 lines were used and described in Flybase except when noted: *ptc-Gal4*, *dpp-Gal4*, *rn-Gal4*, *Dll^212^-Gal4*, *ap^42B11^-Gal4 and dysf-Gal4* (Flylight database: http://flweb.janelia.org/cgi-bin/flew.cgi) (Jory et al., 2012), *hh-Gal4*, *bib-Gal4*. To temporarily restrict the activity of the different Gal4 lines we used the tubulin-Gal80ts system. Briefly, embryos were collected for 24 to 48 h, maintained at the restrictive temperature (17°C) and then shifted to the permissive temperature (31°C) for the required time before dissection.

The following UAS lines were used: *UAS-GFP*, *UAS-cherry-RNAi* [used as control, Bloomington *Drosophila* Stock Center (BDSC), #35785], *UAS-MMP2* (BDSC, #58705), *UAS-dysf-RNAi* [Vienna *Drosophila* Resource Center (VDRC), #110381], *UAS-CycE-RNAi* (VDRC, #110204), *UAS-E2f1-RNAi* (VDRC, #108837), *UAS-Cdk1-RNAi* (VDRC, #106130 and BDSC, #28368), *UAS-E2f1+Dp* (BDSC, #4774 and #4770), *UAS-miRHG* (Siegrist et al., 2010), *UAS-yki* (Zhang et al., 2008), *UAS-Rbf^280^* (BDSC, #50748), UAS-*Rho1-BD-GFP* (Simoes et al., 2006) and *UAS-dysf* (Jiang and Crews, 2003). Some of the RNAi lines were combined with UAS-Dcr2 (BDSC, #24646) to enhance its effect. Other reporter and GFP protein trapped lines used were: *E-cad-GFP* (BDSC, #60584), *vkg-GFP* (FlyTrap, G205), *bib-lacZ* and *E(spl)-mβ-CD2* (de Celis et al., 1998), *dysf-lacZ* (Cordoba and Estella, 2014), *E2f1-GFP* (BDSC, #83388), *ZCL2207 Atpα-GFP* (*Drosophila* Genetic Resource Center, Kyoto, #110860) and *sqh-GFP* (gift from M. Suzanne, CBI, Toulouse, France).

A combination of a null allele (*Cdk1^null^*; BDSC, #6643) and a temperature-sensitive allele of *Cdk1* (*Cdk1^E1–24^*; BDSC, #6641) was used. Briefly, embryos from the genotype *Cdk1^null^*, *UAS-dysf/Cdk1^E1-24^; dpp^Gal80^>GFP* and *UAS*-*dysf/+*; *dpp^Gal80^>GFP* were collected for 24 to 48 h, maintained at 17°C and then shifted to 31°C to inactivate Cdk1 function and induce *dysf* expression for 24 h before dissection.

To generate single cell clones we used the line *yw hs-flp; LexAop-FRT-stop-FRT-CD8::GFP* (BDSC, #57588) to label cell membranes. An 8-min heat shock at 37°C was applied to late third instar larvae and white pupae and dissected at the indicated times.

### Immunostaining

Standard procedures were used to fix and stain prepupal and larval leg and wing imaginal discs (detailed below). The prepupal stage is considered to be between 0 and 12 h APF.

Briefly, larvae and prepupae were dissected in PBS and fixed with 4% paraformaldehyde in PBS for 25 min at room temperature. They were blocked in PBS, 1% bovine serum albumin, 0.3% Triton X-100 for 1 h, incubated with the primary antibody overnight at 4°C, washed four times in blocking buffer, and incubated with the appropriate fluorescent secondary antibodies for 1.5 h at room temperature in the dark. They were then washed and mounted in Vectashield (Vector Laboratories, #H-1000). We used anti-Phalloidin (TRITC) (Sigma-Aldrich, #P1951) to stain the actin cytoskeleton and TOPRO (Thermo Fisher Scientific, #T3605) or Dapi (Merck) to stain nuclei.

As primary antibodies, we used mouse anti-Dlg [Developmental Studies Hybridoma Bank (DSHB), #4F3; 1:50], rat anti-Ser (a gift from Ken Irvine, Rutgers University, NJ, USA; 1/1000), mouse anti-En (DSHB, #4D9; 1:50), rabbit and mouse anti-β-Gal (MP Biomedics, 559761 and Promega, #Z378A; 1:1000), rabbit and mouse anti-pH3 (Merck Millipore, #06-570 and Cell Signaling Technology, #9796: 1/500), rat anti-CD2 (Serotec, #MCA444R; 1/200) and rat anti-Ci (DSHB, 2A1; 1/50).

To preserve the 3D structure of the imaginal discs, the samples were mounted using an adhesive spacer at both sides of the samples in the slide. A coverslip was then placed on top of the spacer.

When indicated, prepupae were synchronized to properly compare fold formation phenotypes. White pupae of the given phenotype were selected, incubated for 0, 3, 5 and 6 h at the required temperature and then dissected and stained following standard procedures (see below).

For EdU staining, dissected leg discs were cultured in 1 ml of EdU labeling solution for 20 min at room temperature and subsequently fixed in 4% paraformaldehyde for 30 min at room temperature. EdU detection was performed according to the manufacturer's instructions (Click-iT EdU Alexa Fluor Imaging Kit, Thermo Fisher Scientific) and leg discs were incubated for 30-40 min at room temperature in the dark. All confocal images were obtained using a Leica LSM710 vertical confocal microscope and a confocal spinning disk Olympus SpinSR10 and were treated using Fiji and Adobe Photoshop programs.

### Live imaging of prepupal leg imaginal discs

Leg joint formation movies were taken as in Guarner et al. (2014) with small modifications. White prepupa from the Atpα-GFP line (to label cell membranes) were washed in 1× PBS and dissected in Schneider's medium (11720-034, Invitrogen), supplemented with 15% fetal bovine serum (10106-169, Invitrogen) and 0.5% penicillin-streptomycin (15140-122, Invitrogen) and transferred to 35 mm glass-bottom culture dishes coated with poly-D-lysine (P35GC-1.5-10-C, Mattek Corporation) with more Schneider's medium. To reproduce *ex-vivo* imaginal disc eversion, 20-hydroxyecdysone (H5142, Sigma-Aldrich) was added at a final concentration of 0.1 μg/ml. Methylcellulose (M0387-100G, Sigma-Aldrich) was used at a final concentration of 0.3% to maintain leg discs in place. Time-lapse movies were obtained using an inverted confocal microscope (IX83 Olympus) coupled to a confocal spinning disk SpinSR10 (Olympus) at room temperature. Images were taken every 8 min for 6 h. Movies were mounted and analyzed with Fiji/ImageJ software.

### Quantification and statistical analysis

The reduction in the number of cells in the distal domain of the leg after knocking down E2f1 for 48 h (*hh^Gal80^>E2f1-RNAi*) was performed using a program developed in-house (Ledesma-Terrón et al., 2021 preprint) that segments and localizes individual nuclei in a crowded three-dimensional confocal reconstruction. Briefly, the tool segments the nuclei in each plane and then uses a combination of nonlinear fitting algorithms and statistical analysis to identify all confocal sections for each stained nucleus. Next, the centroid of each identified nucleus is labeled according whether it is inside or outside the domain marked by GFP staining. The values of the total number of nuclei in each region are then computed to establish the differences in proliferation between the two regions.

The mitotic index was calculated as the number of pH3-positive cells per area or volume, depending on the experiment. The severity of the phenotypes in Fig. 2 is assessed by counting the number of tarsal joints that presented folding defects: ‘not lost’ if the tarsal folds are not affected; ‘partially lost’ when the tarsal fold is recognized but reduced compared with the corresponding control one; ‘lost’ when the tarsal fold is not present. Sample size is indicated in each figure legend.

Rho1-BD-GFP relative fluorescence intensity was quantified by measuring the mean intensity of cells in the fold and interjoint domains and their areas were calculated manually using the ROI measurement tool in Fiji. Apical F-actin fluorescence intensity was measured in the joint domain cells or in the corresponding domain in the experimental compartment using Fiji. Sagittal images were obtained for prepupal leg imaginal discs.

Tissue height was quantified using sagittal and cross-sectional images from prepupal leg and imaginal discs, respectively, and stained with Phalloidin. Cross-sectional images from wing imaginal discs were obtained by Fiji from multiple *z*-stacks that cover the whole disc. As the main epithelium of the wing and leg imaginal discs is formed by a monolayer of cells, the height of the tissue could be extrapolated to the relative height of a cell in that specific domain. To do this in the leg disc, we measure the distance from the basement membrane of the epithelium to the highest point in the interjoint domain (proximal to *dysf*) and to the lowest point in the joint domain (distal to *dysf*) (see Fig. 4A). In the wing disc we measured the distance from the base of the epithelium to the deepest point in the region of interest or fold (see Fig. 6D). The depth of the fold was determined by measuring the distance from the highest adjacent points of the tissue to the deepest point of the fold region using a perpendicular line, as shown in Figs 4 and 6. To avoid errors the 4/5 tarsal fold was excluded from the quantifications, except when explicitly indicated, as it is the last to form.

Statistical analysis was performed using the GraphPad Prism software (https://www.graphpad.com). To compare between two groups, a non-parametric Student's *t*-test was used. To compare between more than two groups, a non-parametric one-way ANOVA Dunnett's test was used. Sample size is indicated in each figure legend.

### Adult leg preparations

Adult or pharate (in the case of flies that could not hatch) legs of the required phenotypes were collected in 96% ethanol until mounted. We used Hoyer's mounting medium in a 1:1 proportion with lactic acid (90%, Merck) to preserve the cuticle of the legs. Multiple focal planes of each leg were acquired and then combined using the Helicon Focus program to create a fully focused image of the legs.

#### Simulation of leg epithelial folding

For the simulations, we developed a simplified agent-base model written in Julia language as a Jupyter notebook (https://github.com/davidgmiguez/leg), using Plots (for illustrations) and RollingFunctions (for smoothing and average functions). In the model, cells are numerical entities with spatial dimensions defined by the user, organized as a one-dimensional array that simulates a monolayer, embedded in a domain of fixed length. The model is a time-based algorithm with a for cycle that sets the total number of iterations and the time-step. For each iteration, a single cell in the tissue is chosen at random, that is set to grow and/or proliferate based on the following rules. (1) Cells grow by expanding any of the four vertices. The direction of growth is chosen at random. As the domain size is fixed, cells grow by compressing their neighboring cells, therefore, to maintain the integrity of the tissue, when a given cell is set to grow in a given direction, the adjacent cell is compressed accordingly. (2) A minimal cell size is defined in such a way that a cell cannot be compressed below this value. Consequently, a cell selected to grow in the close vicinity has to expand the other way. (3) Two types of cells are defined: cells that can grow both horizontally and vertically (labeled in blue) and cells that, due to elevated levels of actin, cannot grow vertically (labeled in red). Color intensity of the cells illustrates the compression level of each cell (light color correspond to large uncompressed cells, and dark color corresponds to a cell highly compressed close to its minimal size). Therefore, red cells close to a highly compressed cell tend to expand apically, whereas blue cells set to grow against a highly compressed neighbor expand basally. (4) The cell cycle length (defined as the number of iterations for a cell to enter mitosis and divide) is set by the user. When a given cell is older than this value, it divides in half, producing two new cells of the same type, that occupy the same space as their mother.

The state of the system in terms of size and position of each cell is recorded at each time step and drawn to generate the time-lapse movies for each condition.

## Supplementary Material



10.1242/develop.202384_sup1 Supplementary information
