# Supplementary material for: Cell proliferation and Notch signaling coordinate the formation of epithelial folds in the Drosophila leg
Source: Development. 2024 Apr 16;151(8):dev202384. doi: 10.1242/dev.202384 (PMC11058088; doi:10.1242/dev.202384)
Supplement: Supplementary information [file develop-151-202384-s1.pdf]

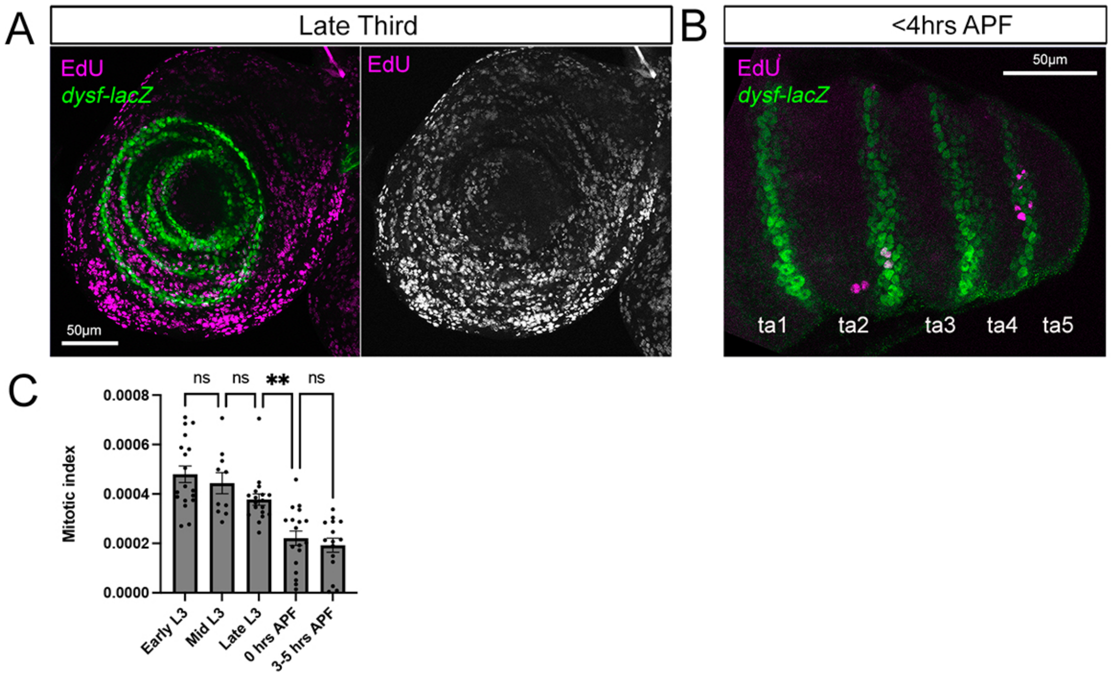

Fig. S1. Cell proliferation and *dysf* expression during leg development.

A and B) Third instar (A) and distal prepupal (B) leg imaginal disc stained for EdU and *dysf-lacZ*. ta, tarsal segment.

C) Mitotic index calculated as the number of pH3 positive cells per leg imaginal disc volume at different times of development. Note the reduction of proliferating cells as the leg disc develops. Statistical analysis by t-test of the comparisons indicated. **<p=0.01, and ns=not significant.

Development • Supplementary information

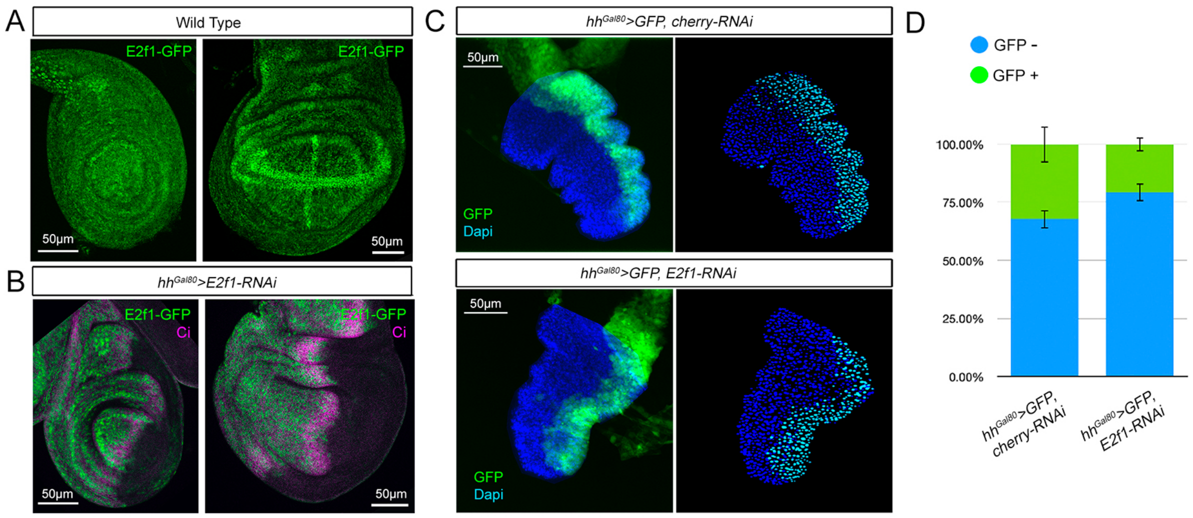

Fig. S2. Downregulation of E2f1 in leg and wing imaginal discs.

A) *E2f1-GFP* (green) expression in third instar leg and wing imaginal discs.

B) Leg and wing imaginal discs of the *hh*^{Gal80}>*E2f1-RNAi* genotype dissected 48 hrs after inducing the transgene in the posterior compartment and stained for E2f1-GFP and Ci.

C) Distal domain of prepupal leg imaginal discs expressing the indicated transgenes in the posterior compartment (*hh*^{Gal80}>) for 48 hrs and stained for GFP and Dapi. Also shown is the segmented image used for the quantification of cell numbers.

D) Quantification of the cell number reduction in the distal part of prepupal legs for the genotypes indicated as in C. *hh*^{Gal80}>*GFP, E2f1-RNAi* (n=4) and *hh*^{Gal80}>*GFP, cherry-RNAi* (n=4). Cell numbers in the GFP+ and GFP- domains are represented as % of the total cell numbers in the distal leg domain. Error bars represent the standard deviation.

Development • Supplementary information

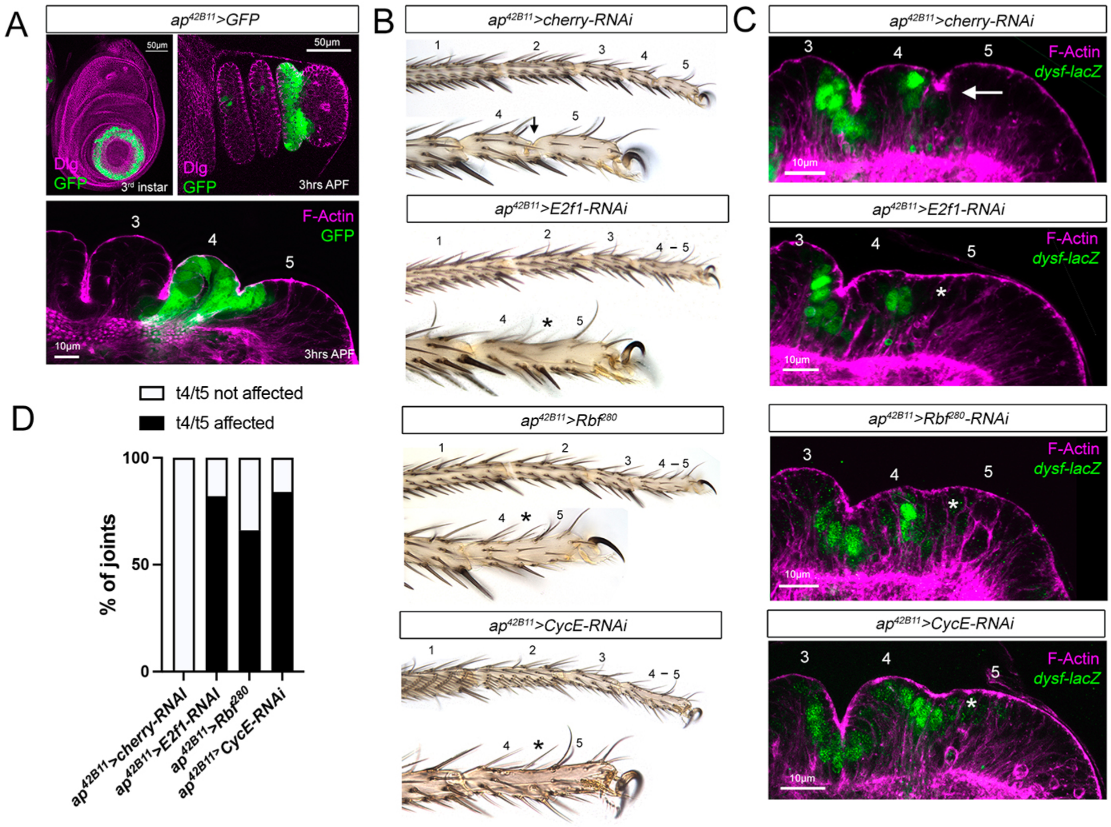

Fig. S3. Fold and adult joint phenotypes after reducing cell proliferation in the leg.

A) Third instar leg and prepupal leg imaginal discs stained for Dlg or F-actin and the *ap*^{42B11}*>GFP* line. A higher magnification of a sagittal view of the last tarsal segments showing the Ap domain that encompasses the fold between the fourth and fifth tarsal segments.

B) Adult legs expressing the indicated transgenes with the *ap*^{42B11}*>* driver. The tarsal segments and a higher magnification of the last segments is shown. An arrow points to the ta4/ta5 joint and an asterisk indicates the absence of the joint.

C) Distal region of 3-4 hrs APF leg discs stained for F-actin and *dysf-lacZ* expressing the indicated transgenes with the *ap*^{42B11}*>* driver. The fold between the fourth and fifth tarsal segments is indicated with an arrow and its absence or defective formation with an asterisk.

D) Quantification of the ta4/ta5 adult joint phenotypes for the genotypes indicated. The number of adult legs scored are: *ap*^{42B11}*>cherry-RNAi*: 48, *ap*^{42B11}*>E2f1-RNAi*: 65, *ap*^{42B11}*>Rbf*^{280}: 53 and *ap*^{42B11}*>CycE-RNAi*: 57.

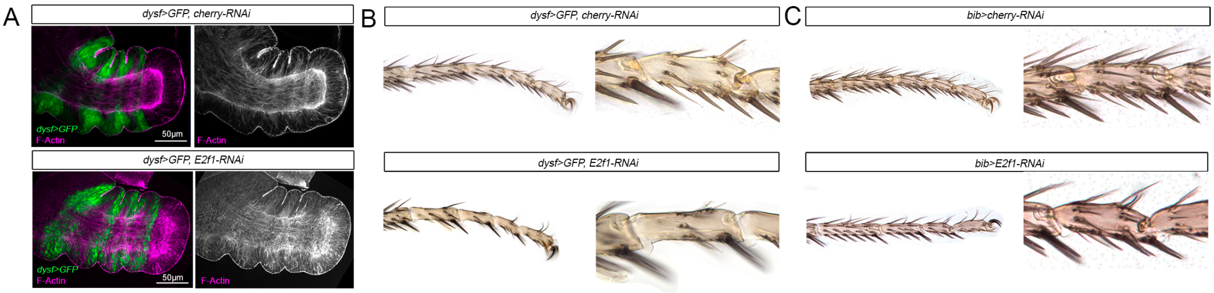

Fig. S4. Knocking down E2f1 in the joint domain.

A) Tarsal region of prepupal leg imaginal discs expressing the indicated transgenes with the *dysf-Gal4* line stained for F-actin and for GFP.

B) Tarsal segments from adult's legs of the genotypes in A, expressing the corresponding transgenes. We observed the lack of some bristles in the tarsal segments due to the late residual activity of the *dysf-Gal4* line in the interjoint domain and the requirement of cell division in the sensory organ precursors.

C) Tarsal segments from adult's legs expressing the indicated transgenes with the *bib-Gal4* line.

Development • Supplementary information

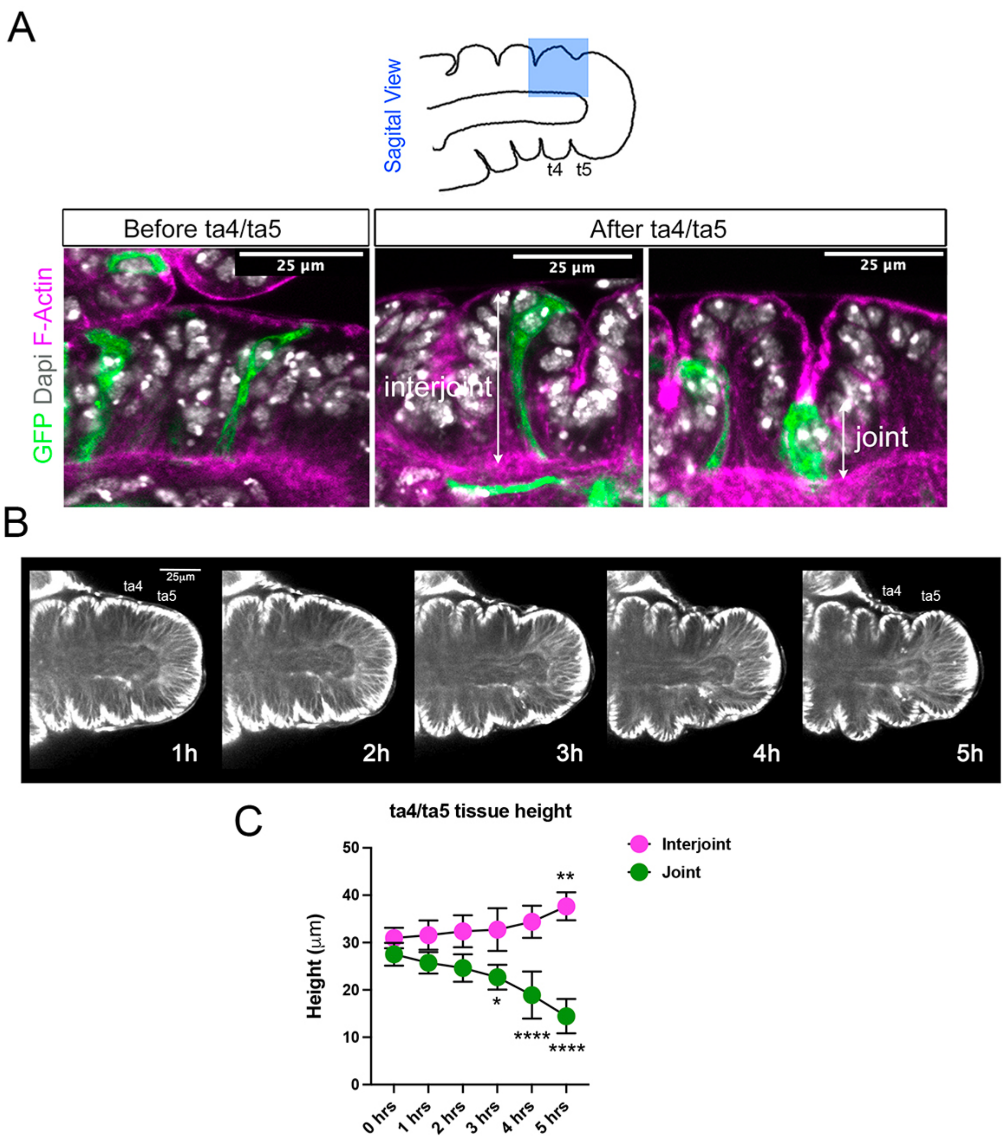

Fig. S5. Cell shape changes and live imaging during the formation of the ta4/ta5 fold.

A) Cell shape changes observed during the formation of the 4/5 tarsal fold in prepupal leg imaginal disc stained for F-actin and Dapi. To visualize single cells, flip-out clones were generated that expressed the membrane tagged CD8-GFP (see methods). Note the different apico-basal height of interjoint and joint cells after the formation of the fold.

B) Still images from a live image movie captured on a prepupa leg during the formation of tarsal folds 4 and 5.

C) Quantification of tissue height at the ta4/ta5 interjoint and joint domains obtained from live images of 8 prepupal legs from 0 hrs to 5 hrs APF. Error bars represent SD. Statistical analysis by one-way ANOVA when compared the mean of each time point with the mean of the control (0 hrs) as indicated. *<p=0.05, ***<p=0.001 and ****<p=0.0001.

Development • Supplementary information

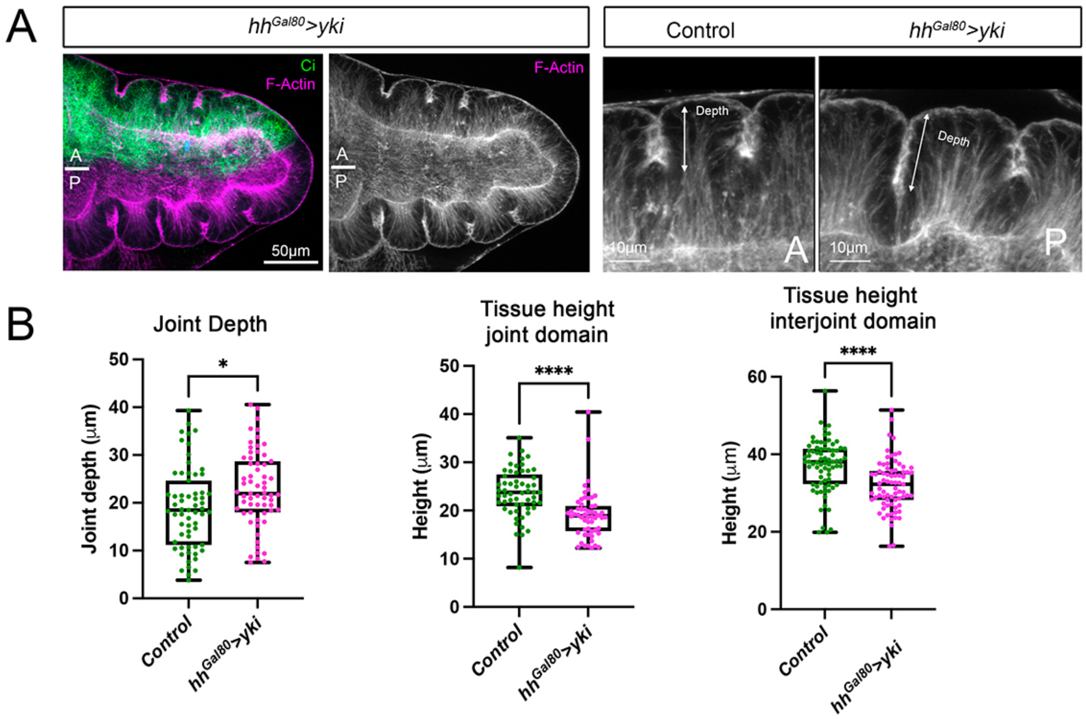

Fig. S6. Temporal expression of *yki* in the posterior compartment.

A) Prepupal leg imaginal discs of the hh^{Gal80}>*yki* genotype dissected 24-30 hrs after inducing the transgene in the posterior compartment and stained for F-actin and Ci. The antero-posterior compartment boundary is represented by a white line. A higher magnification of anterior (A) and posterior (P) tarsal folds is indicated. The anterior compartment is used as a control. Also indicated is how the joints depth is measured.

B) Quantification of joint depth and of tissue height at the interjoint and joint domains of the prepupal legs in A. A total of 19 legs were dissected and 56 joints and 75 interjoints were measured. ****$p<0.0001$ and *$p<0.05$ with Student's t test, indicating a significant difference from control. Error bars represent the minimum and maximum points.

Development • Supplementary information

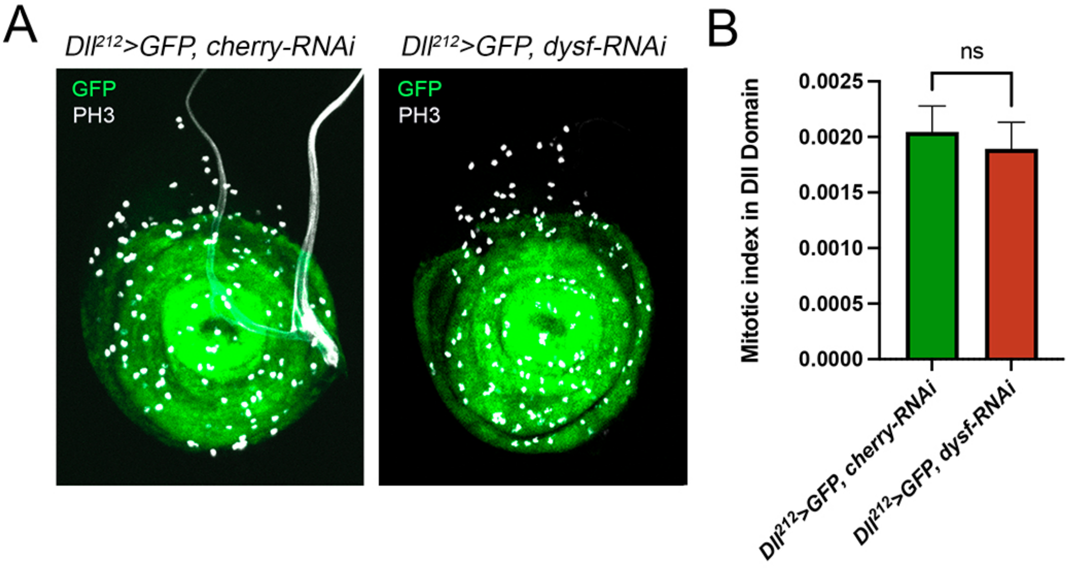

Fig. S7. Cell proliferation is not affected in the absence of Dysf.

A) Downregulation of Dysf in the distal domain of the leg (*Dll*^{212}*>GFP, dysf-RNAi*) does not reduce the number of mitotic cells as visualized by pH3 staining in third instar leg imaginal discs when compared to the control (*Dll*^{212}*>GFP, cherry-RNAi*).

B) Mitotic index calculated as the number of pH3 positive cells in the Distal-less (Dll) domain of the leg (green) of the genotypes described in A. At least 15 legs were analyzed for each condition. Statistical analysis by t-test of the comparisons indicated. ns=not significant.

Development • Supplementary information

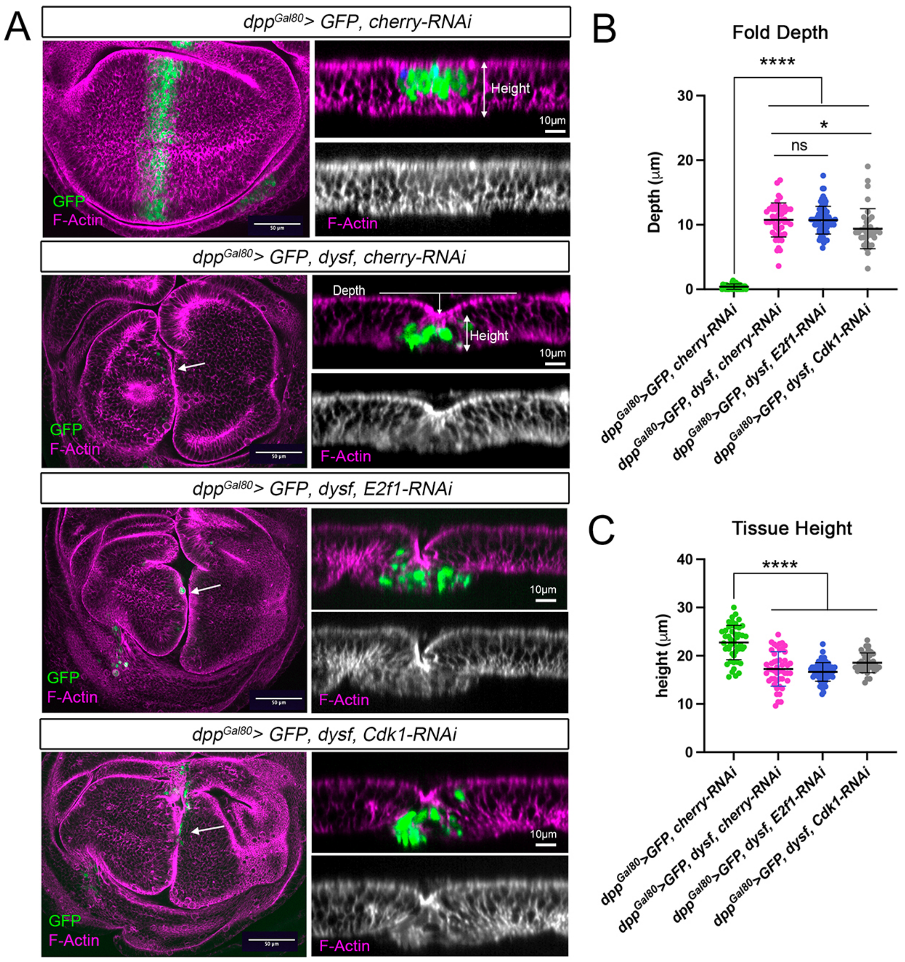

Fig. S8. Fold induction by Dysf in cell cycle arrested cells.

A) Apical view and Z-section the pouch region of wing imaginal discs expressing the indicated transgenes for 48 hrs under the dpp^{Gal80} line. The *dpp-Gal4* driver was used to express *GFP* and *dysf* and RNAis against cherry (control), E2f1 or Cdk1 in a band of cells of the anterior compartment of the wing pouch. F-actin and GFP. The fold generated by the ectopic expression of *dysf* is marked by an arrow. Also indicated is how the fold depth and tissue height are measured.

B and C) Quantification of fold depth (B) and tissue height (C) in the GFP domain measured in the Z-sections of the genotypes described in A. dpp^{Gal80}*>GFP, cherry-RNAi* (n=16), dpp^{Gal80}*>GFP, dysf, cherry-RNAi* (n=24), dpp^{Gal80}*>GFP, dysf, E2f1-RNAi* (36) and dpp^{Gal80}*>GFP, dysf, Cdk1-RNAi* (n=18). Two measurements were done for each disc at different locations. Statistical analysis by one-way ANOVA when compared the mean of each genotype with the mean of the control as indicated. ***<p=0.001, *p<0.05 and ns=not significant.

Development • Supplementary information

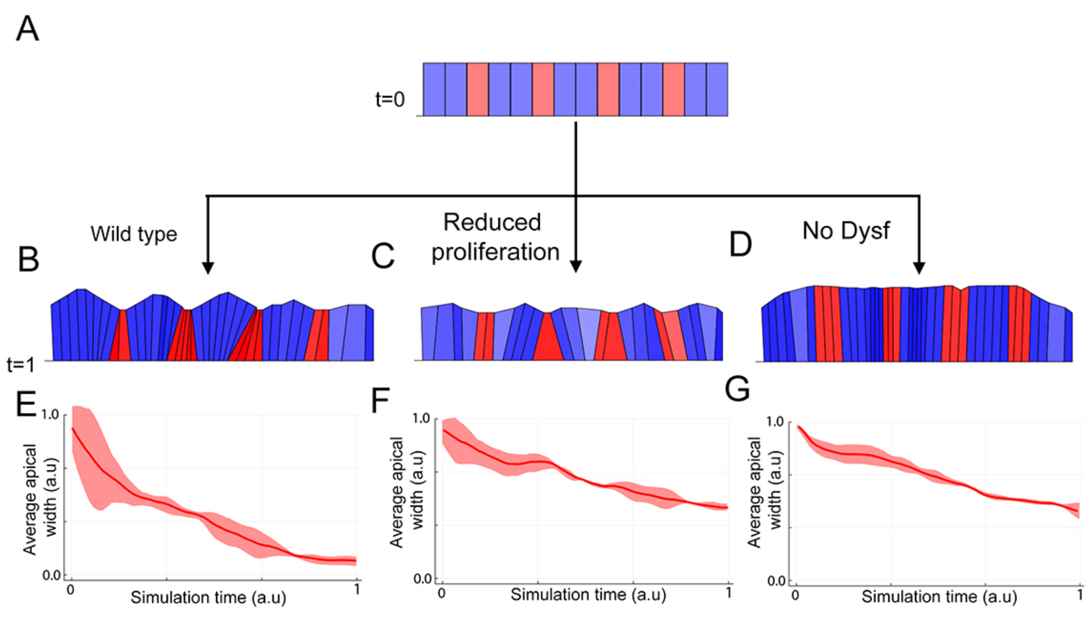

Fig. S9. Simulation of leg epithelial folding and apical width measurements.

A) Initial stage (t=0) of the mathematical simulation of the early events of tarsal epithelial folding. The different colors indicate the interjoint (blue) and joint (red) domains.

B-D) Final stage of the simulation (t=1) after allowing the cells proliferate normally (B), by reducing proliferation (C) and by removing the restriction to grow apically (D). See the text for full details.

E-G) Quantification of the average apical width of the red cells for each experimental condition of the simulation.

Development • Supplementary information

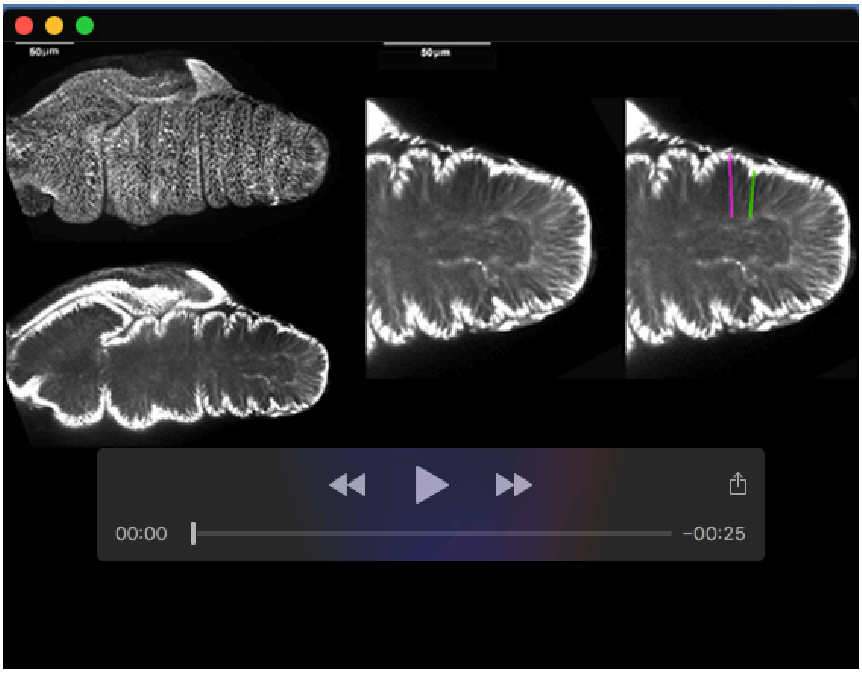

Movie 1. Live imaging of ta4/ta5 fold formation

Live imaging of prepupal leg imaginal disc to visualize the formation of the ta4/ta5 leg epithelial fold from 0 to 5 hrs APF. Cell membranes are marked with Atpα-GFP. Apical and sagittal views are shown for the whole leg. A close-up sagittal view of the ta4/ta5 fold is shown where the relative height of the interjoint and joint domain are marked with colored lines.

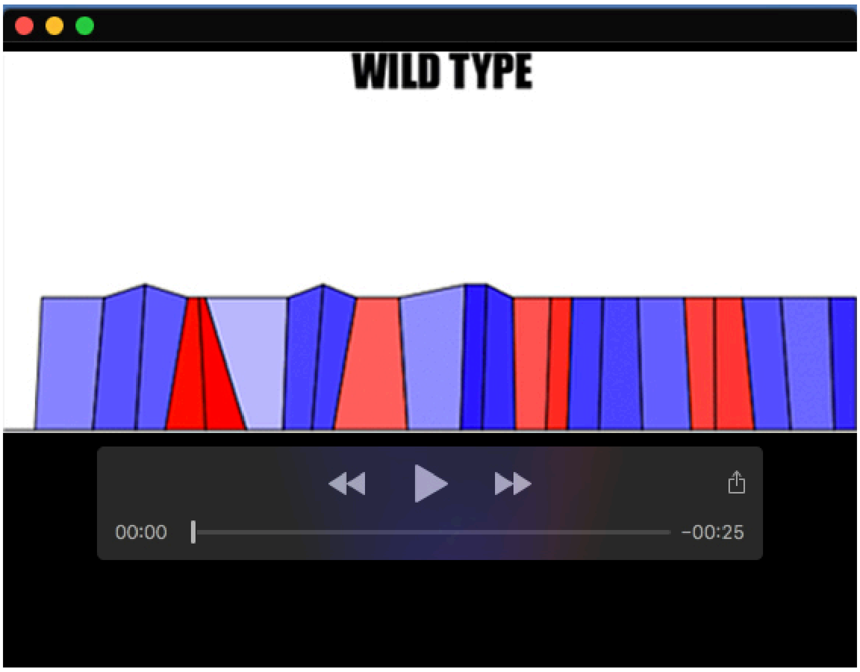

Movie 2. Giff movie representing the computer-based simulation of the early events of tarsal epithelial folding. The different colors indicate the interjoint (blue) and joint (red) domains.

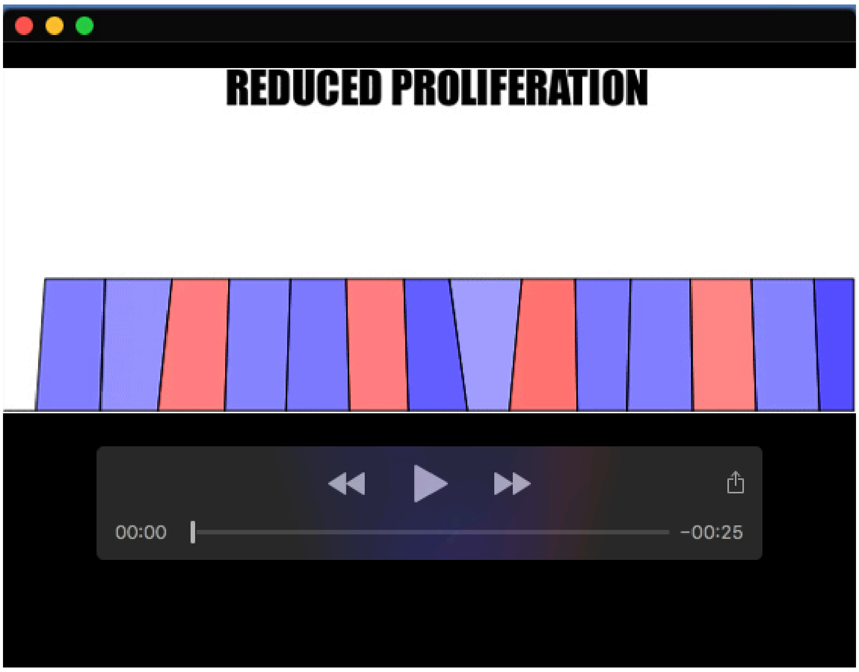

Movie 3. Giff movie representing the computer-based simulation of the early events of tarsal epithelial folding after reducing cell proliferation. The different colors indicate the interjoint (blue) and joint (red) domains.

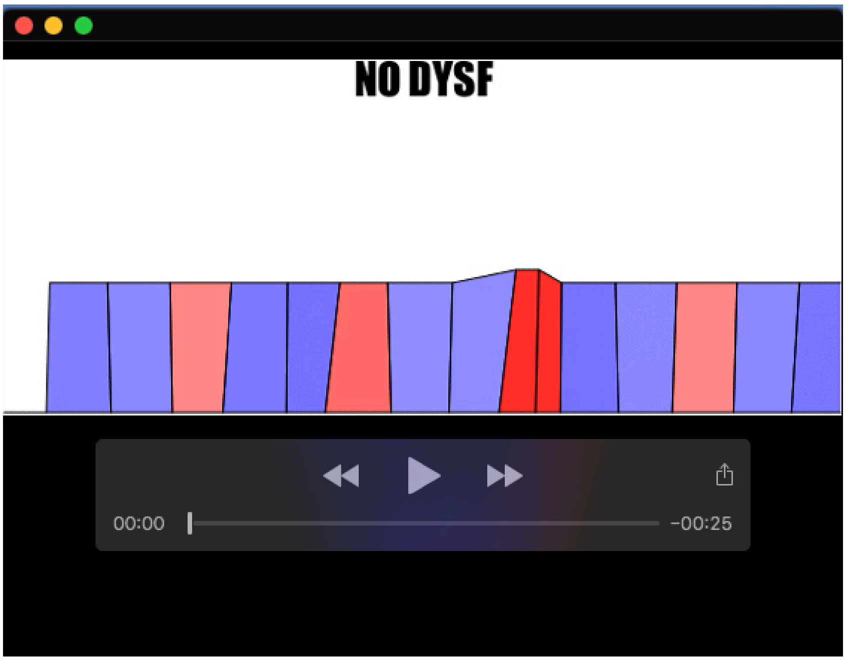

Movie 4. Giff movie representing the computer-based simulation of the early events of tarsal epithelial folding after removing the restriction to grow apically as it happens in a *dysf* mutant. The different colors indicate the interjoint (blue) and joint (red) domains.

Development • Supplementary information
