# Supplementary material for: Cell proliferation and Notch signaling coordinate the formation of epithelial folds in the Drosophila leg
Source: Development. 2024 Apr 16;151(8):dev202384. doi: 10.1242/dev.202384 (PMC11058088; doi:10.1242/dev.202384)
Supplement: Supplementary file 1 [file develop-151-202384_review_history.pdf]

Cell proliferation and Notch signaling coordinate the formation of epithelial folds in the *Drosophila* leg

Alonso Rodríguez, David Foronda, Sergio Córdoba, Daniel Felipe-Cordero, Antonio Baonza, David G. Miguez and Carlos Estella
DOI: 10.1242/dev.202384

Editor: Cassandra Extavour

Review timeline

Original submission:	28 September 2023
Editorial decision:	27 November 2023
First revision received:	6 February 2024
Accepted:	12 March 2024

Original submission

First decision letter

MS ID#: DEVELOP/2023/202384

MS TITLE: Cell proliferation and Notch signaling coordinate the formation of epithelial folds in the Drosophila leg

AUTHORS: Alonso Rodriguez, Sergio Cordoba, Daniel Delipe-Cordero, Antonio Baonza, David Miguez, and Carlos Estella

Please accept my sincere apologies for the long delay in returning these reports to you and a decision on your submitted MS. This is not at all a reflection on your MS or on the reviewer responses to it, but rather a consequence of my own excessive commitments in recent weeks. This is not something that should trickle down to you and your co-authors, and I am sorry for this, as it is not the experience that we want authors such as yourselves to have when you submit your work to Development.

I have now received all the referees' reports on the above manuscript, and have reached a decision. The referees' comments are appended below, or you can access them online: please go to BenchPress and click on the 'Manuscripts with Decisions' queue in the Author Area.

As you will see, the referees express considerable interest in your work, but have some significant criticisms and recommend a substantial revision of your manuscript before we can consider publication. If you are able to revise the manuscript along the lines suggested, which may involve further experiments, I will be happy receive a revised version of the manuscript. Your revised paper will be re-reviewed by one or more of the original referees, and acceptance of your manuscript will depend on your addressing satisfactorily the reviewers' major concerns. Please also note that Development will normally permit only one round of major revision. If it would be helpful, you are welcome to contact us to discuss your revision in greater detail. Please send us a point-by-point response indicating your plans for addressing the referees' comments, and we will look over this and provide further guidance.

Please attend to all of the reviewers' comments and ensure that you clearly highlight all changes made in the revised manuscript. Please avoid using 'Tracked changes' in Word files as these are lost in PDF conversion. I should be grateful if you would also provide a point-by-point response detailing

© 2024. Published by The Company of Biologists under the terms of the Creative Commons Attribution License (https://creativecommons.org/licenses/by/4.0/).

how you have dealt with the points raised by the reviewers in the 'Response to Reviewers' box. If you do not agree with any of their criticisms or suggestions please explain clearly why this is so.

Reviewer 1

Advance summary and potential significance to field

The present manuscript by Rodriguez et al. focuses on morphogenesis in leg discs during late larval and early pupal stages in Drosophila. The authors extensively use immunohistochemical techniques, in combination with a variety of genetic manipulations, to elaborate on the formation of tissue folds between the adjoining segments of the fly leg. Overall, the manuscript consists of clear experiments and provides exciting insights into an understudied area of leg disc epithelial morphogenesis. With its comparison of leg disc and wing disc formation, the manuscript notably bridges gaps between existing and new systems to study epithelial morphogenesis, and the implementation of simple simulations further strengthens the tantalizing idea of unifying principles of morphogenesis across spatial and temporal scales.

The manuscript has a clear potential to provide a seminal contribution in establishing the leg disk as a model system to understand epithelial folding as an interplay of genetic and mechanical cues.Throughout results and in the discussion, the authors make a clear attempt at putting their results in the broad context of morphogenetic movements that are well described in other contexts, such as gastrulation in early embryos.

They conclude that their findings highlight the role of tissue-wide forces in shaping a three-dimensional organ in a reproducible manner, with the main idea being that cell divisions in the imaginal disc generate compressive stresses, which in turn contribute to epithelial folding by forcing it to buckle out of plane.

While consistent with the results, the conclusions made by the authors may not always be the most stringent interpretation of the presented results. Before the manuscript can be accepted, there are a few points that would need to be addressed.

Comments for the author

Major points.

1) The authors suggest that an active indentation at the leg segments results in formation of folds. However alternative explanations/ hypotheses for the presented data seem equally possible. Rather than indentation through fold formation, proliferation at the inter-joint regions may, e.g., “puff-up” the regions in between while joint regions are not extensively remodeled. Presence of F-actin at the joint regions can provide the necessary tensile resistance to maintain the tissue dimensions in the joint region, while the inter-joint region proliferates and expands. The end result will look the same in both cases, although through completely different mechanisms. The experiments currently presented are insufficient to distinguish between the two possibilities. To resolve this, the authors could present, e.g., statistics on cell numbers and cell dimensions.
For instance, if indeed cells at the joint region are constricting, then we should see a reduction in cell height while that in the inter-joint region might increase or remain the same.

2) Another alternative explanation/hypothesis for the presented data comes from the observation that there is a rather extensive change in the overall shape of the leg disc. Based on the images presented in the manuscript, it appears that the radius of dysf expressing cell domain shrinks, especially in case of ta1 (Fig. 1 B, C). In such a scenario, it seems also possible that cells intercalate within this domain, and perhaps also in other domains. Such intercalation could also lead to tissue expansion, as the same number of cells try to fit into a smaller domain, and could explain the domain folding. Thus, cell intercalation within inter-joint regions could also lead to the “puffing-up” of the inter-joint regions. The authors may want to consider such mechanisms as an alternative to cell divisions, and they also may want to comment on whether/how much intercalation could in principle contribute to the formation of epithelial folds.

© 2024. Published by The Company of Biologists under the terms of the Creative Commons Attribution License (https://creativecommons.org/licenses/by/4.0/).

3) It appears that the majority of proliferation occurs prior to formation of the epithelial folds, as the folds themselves are most apparent during pupal stages. It is, however, not clear how authors exclude the possibility that the fold formation is a result of change in tissue dimensions, rather than proliferation. For instance, the authors need to exclude that tissue extrinsic compression from, say, the dynamics in the peripodial membrane, causes the fold formation. In case of such extrinsic stresses, we can also have folds or no folds depending on how many cells existed at the larval stage, before the compressive forces were applied during pupal stage. In this sense, cell proliferation might indeed play a role in fold formation, though only indirectly, as the folds might not form in the absence of extrinsic compression despite prior cell proliferation.
It would be helpful if the authors could clarify this point.

4) Assuming the authors can demonstrate that active indentation at the leg segments results in formation of folds (see point 1), the term 'buckling' still does not seem to be appropriate. The authors see that leg disc (and also wing disc) formation in Drosophila requires cell division in the disc itself. They interpret the need for cell division as the need for tissue expansion, which builds up compressive stress that eventually helps the disc to "fold up" in a stereotypical manner. From the manuscript, it reads such that the position of the "folds" are determined by genetic pre-patterning, and that folds never appear outside of the area where they are supposed to form. This phenomenon might better be called something like 'compression-aided folding', since buckling is typically described as a rather sudden and spatially stochastic event of material property failure. For this, data on dynamics of the process is required, which is admittedly difficult to achieve in fixed tissue.
Additionally, leg/wing disc development occurs on rather long time scales, indicating that the build-up of compressive stresses is rather slow, and would also lead to a slow relaxation of the same through gradual tissue deformation and remodeling. To give a few examples to explain this sentiment:

4.a) p.4 The authors say: "From mid-third instar larval stage to prepupa, we observed a sequential accumulation of F-actin in ring-like structures at the folding points, which ultimately form the joints separating the leg segments in the adult stage (Fig. 1A)".

To get a sense of the dynamics here: how long does this developmental window last? These are hours, aren't they? It would be helpful to clarify this so that the reader understands that the terms "folding", "apical constriction", "buckling", etc run on a different time scale. The authors do a good job of introducing these processes in various epithelial contexts in the introduction section, but they also need to highlight the differences in the time scales.

4.b) p.6 The authors say: "We observed that posterior cells at the presumptive joint fold failed to properly constrict apically and show significantly less apical F-actin than control anterior cells (Fig. 3A, B and E)."

Similar to the point above, it is difficult to see apical constriction being such a slow process, especially since it has been described extensively in the embryo context, where it occurs at a time scale of seconds to few minutes.

4.c) p.8, end of second paragraph: "These results suggest that compressive forces generated by cell proliferation promote the upward buckling of the interjoint epithelium and thus contribute to the formation of the tarsal folds"

"Buckling" would be the case, if, w/o actin and MyoII, there is still deformation. But that does not seem to be the case. While buckling may be defined as a passive deformation due to tissue extrinsic factors (forces), the accumulation of F-actin and MyoII that we see in the manuscript rather indicates active and tissue intrinsic causes. Also, the position of the folding remains stereotypical and is not random, arguing against a stochastic failure of material properties (which is buckling).

4.d) p.8, second-last sentence: "These observations suggest that an increase in tissue compression resulting from cell proliferation increase epithelial folding at specific positions."

© 2024. Published by The Company of Biologists under the terms of the Creative Commons Attribution License (https://creativecommons.org/licenses/by/4.0/).

In contrast to the sentence mentioned in 4.c, this statement is much less contentious. Maybe the authors could generally shift the narrative to be consistent with this phrasing, and away from use of the term ‘bucking’.

5) The authors imply a causal relationship between cell height and tissue compression, see p.14, first new paragraph on the page: “A detailed analysis of the phenotypes resulting from reduced cell numbers uncovers a previously unanticipated behavior of the interjoint cells - their ability to increase in height in response to tissue compression.

Based on the presented data it is difficult to conclude that this is really the case. The implied causal relationship between cell height and tissue compression would require support from live imaging. The authors could either rephrase this, or perform additional experiments to exclude the possibility that change in cell height facilitates tissue deformations - showing this through simulations of leg epithelial folding is not sufficient.

6) Studying epithelial morphogenesis in leg disc formation is, without question, challenging. To highlight the efforts and let the reader appreciate the insights gained through this model, the manuscript would benefit from a better description of inherent limitations in the experimental approach and corresponding caveats in the conclusions. For instance, it does not become clear for a non-expert reader why the authors would not use live imaging data, in spite of being able to express fluorescently labeled proteins. At the same time, live imaging immediately comes to mind as a possibility to exclude various hypothetical possibilities, and strengthen the causal relationships (see alternate hypotheses above). Based on the citations of articles previously authored by some of the authors in this study, the authors have extensive experience in working with leg discs, and readers would benefit from a description of experimental limitations from the authors perspective. Again, as an example for the case of live imaging: if this is technically challenging, then it would help if authors mention it and cite a few precedents.

Minor concerns:
1) It is a bit challenging to raise specific points without line numbers in the manuscript.

2) Please switch to color-blind-friendly pseudo-color combinations in colored image panels.

3) Please add scale bars on all representative images. Needless to say, separate scale bars are not required when different channels of the same image are displayed, or if it is obvious that various images are acquired in the same region with the same dimensions. But, this is not the case in a lot of images. Since no scale bar was shown, we had to make assumptions about the scale in the following figures/panels: Fig. 1B, C, Fig. 3 Fig. 4, Fig. 6A, B, D (cross-section views), Fig. 7, and Supplementary Fig. 1-5. Dimensions are relatively easy to gauge when the reader is experienced in looking at microscopy images, but still: please do not force the reader to make assumptions about the scale in the image panels.

4) p.4 The authors say: “From mid-third instar larval stage to prepupa, we observed a sequential accumulation of F-actin in ring-like structures at the folding points, which ultimately form the joints separating the leg segments in the adult stage (Fig. 1A)”.

It may be better to say this more carefully: increased phalloidin suggests accumulation of actin. Also, please rephrase to highlight that this is a “tissue-scale” ring structure, which is rather distinct from “cellular-scale” F-actin rings seen in cytokinesis.

5) p.4 The authors say: “Dividing cells are found throughout the leg imaginal discs with no particular cell division pattern and their number progressively decreases during early prepupal stages (Fig. 1A and S1 Fig).”

It would help if the authors support their quantitative statement about the decreasing number of dividing cells, with quantification of number of dividing cells. What is the fraction of dividing cells? Is it the absolute number or the fraction of dividing cells that’s more relevant? Is the number/fraction of dividing cells similar in anterior vs. posterior compartment? How about joint vs inter-joint regions? How would these differences/similarities affect the outcome of the hypotheses about the involvement of cell proliferation on fold formation?

© 2024. Published by The Company of Biologists under the terms of the Creative Commons Attribution License (https://creativecommons.org/licenses/by/4.0/).

6) p.5 in the same paragraph the authors say, “However, we did not observe a reduction in the mitotic index in the wing disc, calculated as the number of pH3-positive cells per area, suggesting that cells are proliferating at a slower rate (S2 Fig).”

This statement about the wing disk appears somewhat out-of-the-blue. It is unclear why the student's t-test was performed in S2 Fig.D when the data in E2f1 RNAi is bimodal. Here, a non-parametric test is recommended. Also, the definition of mitotic index could also be included in the methods, if space permits.

7) p.8 authors say, “Next, we analyzed the height of interjoint and joint cells after reducing cell proliferation by expressing the E2f1-RNAi line in the posterior compartment, and compared it with the anterior control in 3 hrs APF leg discs.”

It is not quite clear where to find the reference for the quantification in comparing anterior and posterior compartment. For the quantifications discussed in the results section ‘Tissue compression generated by cell proliferation is necessary for leg epithelial folding’, presented in Fig.4 and S5, it isn’t clear when the measurements are made in the anterior compartment vs. in the posterior compartment. This needs to be clearly explained. If the control condition mentioned in the figure is indeed measurements in the anterior compartment, then it is unclear why the measurements were not compared between a purely genetic outcross control vs. perturbations, as the indentation depth seems to be different between anterior and posterior sides of the indentation even in control leg disks.

Reviewer 2

Advance summary and potential significance to field

The manuscript addresses the role of cell proliferation in driving the formation of folds during tissue morphogenesis using the tarsal fold in the Drosophila leg as a model. The authors find that global proliferation results in compressive forces. These forces coordinate with Notch signaling to result in buckling or folding of the tissue at regions where Rho1 is active in a Notch-dependent manner.
While the idea that global cell proliferation under constraint drives tissue buckling is not novel, coupling this finding with a (Notch) signaling pattern to address the stereotypic nature of (tarsal) tissue folds is a significant advance in the field. The manuscript is well-written, with compelling and elegant experiments. I also appreciate the exhaustive nature of ruling out key mechanisms and testing the applicability of mechanisms in another (wing) tissue.

Comments for the author

Major comments for revision:
1) All images use a red-green combination. Please use a color combination that is accessible for folks with color blindness. A green-magenta combination works well, in my opinion.
2) Figure 3: Rho 1 accumulation in folds and lack thereof in proliferation-deficient and, thereby, fold-deficient tissue is a correlation, not causation. The authors can try to down-regulate Rho1 activity in the folds to examine its role when proliferation is unaffected. This maybe tricky if Rho1 is also involved in proliferation but the experiment may still work if Rho1 is knocked down only in the joint cells.
3) Fig 4: How the authors measured cell height needs to be clarified. It seems inaccurate if the double-headed straight/perpendicular arrow is trying to follow F-Actin. I couldn’t find it in the methods section either. One way to resolve this is by salt pepper labeling of the cell membrane to accurately measure where the boundary of the cells is- the cell's height may be at an angle from the base following the bends (rather than orthogonal from the base). Inferring compressive forces from cell height is also unclear - is the argument that taller cells are compressed while the volume conserved? Apical constriction in joint cells can also create “compressive forces,” resulting in the expansion of interjoint cells (rather than cell proliferation alone).
4) Fig 6A: Is cell proliferation normal in dysf RNAi? If yes, it supports the result in the posterior part, where dysf is downregulated, the tissue is wavy/buckling, albeit abnormally or in a non-

© 2024. Published by The Company of Biologists under the terms of the Creative Commons Attribution License (https://creativecommons.org/licenses/by/4.0/).

patterned way. Relatedly, can the authors express a CA form of Rho in a spatially controlled manner to rescue the dysf RNAi phenotype?

Minor:

1) Fig 1C: It needs to be clarified what is apical and basal. It becomes clear with the BM staining later. Please annotate this figure.
2) Fig 2F: Rbf280 does not look like control in the anterior section, and CyCE tissue looks bigger- can be discussed further.
3) S4A: Are there fewer folds overall? Even in control?
4) Fig 6 D: Measuring cell height with orthogonal lines does not seem accurate as the phalloidin staining is at an angle following the bending of the cells (see major comment 3).
5) Scale bars need to be included in several figures. Please add them everywhere.

Reviewer 3

Advance summary and potential significance to field

In this manuscript, Rodriguez and colleagues investigate the role of Notch signaling and cell proliferation in the formation of epithelial folds. Using the Drosophila leg as a model, they find that effective formation of leg tarsal folds requires a combination of proliferation within the epithelium together with Notch signaling to specify the location of the folds; specifically, the Notch target dysfunction is responsible for producing targeted action of actomyosin within the joint cells to shorten their apical-basal length and causing their apical constriction. The authors propose a mechanistic model requiring the coordinated action of proliferation (within the entire tissue, not merely the joint cells themselves), which creates tissue-wide compression forces, together with dysfunction-dependent control of the height and apical constriction of joint cells, which together produce epithelial buckling at the appropriate locations.

The paper is overall well written, and the authors make a compelling case in the first part of their narrative that (1) a reduction of proliferation results in a flattened epithelium and a loss of tarsal folds (while Notch activity and localization are preserved), and that this effect requires a reduction of global proliferation (both interjoint and joint cells), rather than merely that of joint cells; (2) that in proliferation-deficient cells, Rho1 activity and apical constriction are reduced.

The authors go on to propose that proliferation creates tissue compression, which is measured experimentally through the height increase of the interjoint cells with a concomitant height decrease of the joint cells; the generation of the compression forces requires an intact ECM. In addition, the authors propose that Dysf is directly responsible for Rho1-mediated apical-basal shortening of the joint cells (as well as apical constriction), and follow up with a straightforward computational finite-element model that recapitulates the distinct roles of proliferation (leading to compression) and Dysf-dependent apico-basal height constraint in generating epithelial folding.

In this reviewer's opinion, a strength of this paper was its compelling narrative and good logical flow. One experimental detail that I particularly appreciated was the approach of specifically knocking down the function of specific genes in the posterior compartment, so that control (anterior) and knockdown (posterior) could be directly compared in the same animal. In addition, the mechanistic model - the generation of controlled buckles through a combination of planar compression forces with locally controlled apico-basal asymmetries is new in this particular system and of general interest to the morphogenesis field.

However, I was not as convinced by the data in the second part of the manuscript as in the first part, and I thought that the analysis lacked resolution and rigor in some areas that were critical to making some of the authors' key points.

Comments for the author

MAJOR:

© 2024. Published by The Company of Biologists under the terms of the Creative Commons Attribution License (https://creativecommons.org/licenses/by/4.0/).

(1) The authors make the point more than once that Drosophila leg development is merely one model system for epithelial folding, and that insight into the physical mechanism of epithelial folding in this system will potentially transfer to other systems. While I completely agree, I think it's really unfortunate that the current manuscript is not really written with those 'transfer' readers in mind: I think the current images are hard to understand for any readers other than those who already have a lot of experience with looking at images of Drosophila leg imaginal discs and tarsal folds. In this reviewer's opinion, the introduction section of the manuscript would benefit enormously from a schematic like the one in Figure 8H that shows (a) the layout of the region of the interest within the organism, and (b) especially critically, shows a conceptual drawing of the architecture of the individual cells (with cell-cell junctions and nuclei indicated) within the epithelial tissue, because the cell-cell junctions are very hard to see in the experimental images.

(2) This reviewer finds it disappointing that the entire argument about the relationship between proliferation and compression hinges on measuring a single one-dimensional (and very reductive) parameter, namely the height of the interjoint vs joint cells. Both the scope and Methods description of the current image analysis is very sparse. The authors' mechanistic model about the deformation of the tissue through a combination of proliferative compression with mechanical constraint really begs for live imaging (where individual animals are tracked over sufficient time periods to observe the gradual deformation of the different cell populations with concomitant recruitment of actomyosin and associated tissue bending/folding). I appreciate that performing live imaging experiments may not be entirely feasible, or the authors may plan to do this for their next major project/paper on this topic. However, if the authors' narrative (and the computational model) hinges on the generation of compressive stress, and if they want to make this point convincingly based on immunostaining and fixed images alone, then I would really encourage them to dig deeper into their current data to describe and analyze the organization and re-organization of the cellular architecture during fold formation, and make a convincing attempt at a more robust 2-dimensional description.

Some information that would have helped me interpret and contextualize the current analysis: Can you show explicitly (e.g. by counting nuclei, or segmenting cell-cell junctions) from your current images that cell numbers increase in specific areas over time due to proliferation? Can you show explicitly how these cell numbers are reduced in the phenotypes with reduced proliferation? Can you measure how the total volume of the epithelial sheet changes over these time scales (and thus how cell volumes change during proliferation)? Can you measure – or at least show in processed or segmented image where the cell-cell boundaries are clearly visible – how the distribution of cell volumes (in the apico-basal) direction changes over time? For example, it's a key feature of the current computational model (Figure 8) that interjoint cells expand apically and joint cells expand basally in panel B and C but not in panel D – yet in the accompanying experimental images in Figure 8B,C and D (which are supposed to represent these three different phenotypes) it is absolutely impossible to tell whether that's actually the case.

(3) If I understand correctly, the mechanistic model (incorporated into the computational model) has two required components: Force generation through compression - created by proliferation in combination with some mechanical constraints like the ECM adhesion – plus a programmed symmetry-breaking mechanism, which require dysfunction-dependent mechanical processes in the joint cells to produce the 'seeds' for the buckles. The authors' data seems to suggest that the symmetry-breaking mechanism has two components: One being the constraint (or even active reduction) of the height of the joint cells, the other being the apical constriction of the joint cells, both of which require dysf function and actomysin activity. Based on the results in Figure 3, do the authors have thoughts about whether one or both of these (height control and apical constriction) also require compression forces in order to activate – if so, how (e.g. maybe through mechanosensation of planar force components)? And if so, isn't that a key element that would need to be incorporated into the model? In addition, the computational model makes some key predictions (e.g. in absence of dysf function, we would expect cells to grow indiscriminately higher with proliferation; for increased overall proliferation, cells might start by growing indiscriminately higher with proliferation, and then buckling in random fashion), which are not reflected by experimental findings. I am quite supportive of the inclusion of the computational model, but if the model doesn't accurately reflect the empirical findings, how useful is it? Or is that the indication that some key element is missing that would reconcile those discrepancies?

© 2024. Published by The Company of Biologists under the terms of the Creative Commons Attribution License (https://creativecommons.org/licenses/by/4.0/).

First revision

Author response to reviewers' comments

Dear Cassandra Extavour,

Thank you very much for passing along the reviews of our paper, "Cell proliferation and Notch signaling coordinate the formation of epithelial folds in the Drosophila leg" DEVELOP/2023/202384. We were very excited to receive such thoughtful and favorable comments.

We would like to express our gratitude to you and the reviewers for their positive feedback and valuable suggestions that certainly improve the paper. We have carefully considered all criticisms and made efforts to address each comment by introducing new experiments and incorporating all the suggested changes into the text. We believe that this revised new version of the paper is much improved and will answer all the questions raised by the referees.

Below, you'll find a point-by-point response to all their comments. Additionally, I'd like to highlight some of the new experiments conducted to strengthen our findings.

1) *We have shown that in the leg, cell proliferation generates compressive forces that in coordination with positional clues provided by Notch/Dysf promotes the formation of the leg epithelial folds. Importantly, after reducing cell numbers epithelial folds are not generated despite the presence of Dysf. To validate this result, we have ectopically expressed dysf in the relatively flat epithelium of the wing pouch in a wild type proliferating background and in a cell division deficient background. Notably, and consistent with our results in the leg, fold induction by Dysf depends on cell proliferation as transiently blocking cell division throughout the disc using a temperature sensitive allele of Cdk1 prevents the formation of ectopic folds and the accumulation of F-actin induced by Dysf (see Fig. 6D-F). However, blocking cell proliferation specifically in cells expressing dysf has a minimal effect on its ability to induce a fold (Fig. S8). These results suggests that cell proliferation is required at a tissue-wide level for the induction of the folds, as we have described in the leg.*

2) *In the previous version of the paper, we analyzed whether an increase in cell proliferation is sufficient to induce epithelial folding in the flat epithelium of the wing pouch. To do this we expressed E2f1+Dp, stg+CycE or yki for 48 hrs before dissection using the patched-Gal4, tubGal80ts (ptc^{Gal80}>) line. We failed to observe any ectopic fold in the wing pouch, apart from a small bulge in the epithelium and concluded that an increase of this parameter was not sufficient to start the process on its own.*

   However, when we re-analyzed our data, we realized that in the experiments with ectopic yki expression, we had overlooked the presence of ectopic folds parallel to the D/V boundary of the wing. To validate this observation, we repeated the experiments and confirmed that an increase in cell proliferation is sufficient to induce the formation of epithelial folds, which appear at random positions within the yki-expressing cells (See Fig. 7D-F). We attribute the absence of folds upon overexpression of E2f1+Dp, stg+CycE to the substantial induction cell death under these specific conditions, which compensates for cellular accumulation. This is not the case for the yki expression as cell death is not increased in this experiment. The text has been revised to accurately reflect these observations.

3) *As suggested by the referees, we have made several attempts to live-image the process of fold formation. Live imaging of the leg disc is technically challenging, especially during the transition from the relative flat epithelium of an early third instar disc to the prepupal disc. For this reason, we have focused on the tarsal ta4/ta5 fold, as it is the last one to be formed between 0 and 5 hrs APF. Building upon our previous*

© 2024. Published by The Company of Biologists under the terms of the Creative Commons Attribution License (https://creativecommons.org/licenses/by/4.0/).

observations in fixed tissues, live imaging reveals a dynamic process during fold formation where joint cells undergo apical constriction and reduce their apico-basal size while interjoint cells increase in height in response to tissue compression. We have included this new data and movie in new Fig. S5. Unfortunately, the cellular resolution of the live imaging movies is not good enough to precisely follow the cell shape changes that occur during fold formation as we have done for fixed tissues (Fig. S5).

Reviewer 1 Advance Summary and Potential Significance to Field:
The present manuscript by Rodriguez et al. focuses on morphogenesis in leg discs during late larval and early pupal stages in Drosophila. The authors extensively use immunohistochemical techniques, in combination with a variety of genetic manipulations, to elaborate on the formation of tissue folds between the adjoining segments of the fly leg. Overall, the manuscript consists of clear experiments and provides exciting insights into an understudied area of leg disc epithelial morphogenesis. With its comparison of leg disc and wing disc formation, the manuscript notably bridges gaps between existing and new systems to study epithelial morphogenesis, and the implementation of simple simulations further strengthens the tantalizing idea of unifying principles of morphogenesis across spatial and temporal scales.

The manuscript has a clear potential to provide a seminal contribution in establishing the leg disk as a model system to understand epithelial folding as an interplay of genetic and mechanical cues. Throughout results and in the discussion, the authors make a clear attempt at putting their results in the broad context of morphogenetic movements that are well described in other contexts, such as gastrulation in early embryos. They conclude that their findings highlight the role of tissue-wide forces in shaping a three-dimensional organ in a reproducible manner, with the main idea being that cell divisions in the imaginal disc generate compressive stresses, which in turn contribute to epithelial folding by forcing it to buckle out of plane.

While consistent with the results, the conclusions made by the authors may not always be the most stringent interpretation of the presented results. Before the manuscript can be accepted, there are a few points that would need to be addressed.

We would like to express our gratitude to the reviewer for their positive feedback and valuable suggestions, all of which we have been carefully considered. With this new, improved, and revised version, incorporating the referee's comments and incorporating new experiments, we are confident that it will address all the issues raised.

Reviewer 1 Comments for the Author:
Major points.

1) The authors suggest that an active indentation at the leg segments results in formation of folds. However, alternative explanations/ hypotheses for the presented data seem equally possible. Rather than indentation through fold formation, proliferation at the inter-joint regions may, e.g., "puff-up" the regions in between, while joint regions are not extensively remodeled. Presence of F-actin at the joint regions can provide the necessary tensile resistance to maintain the tissue dimensions in the joint region, while the inter-joint region proliferates and expands. The end result will look the same in both cases, although through completely different mechanisms. The experiments currently presented are insufficient to distinguish between the two possibilities. To resolve this, the authors could present, e.g., statistics on cell numbers and cell dimensions. For instance, if indeed cells at the joint region are constricting, then we should see a reduction in cell height, while that in the inter-joint region might increase or remain the same.

We apologize to the referee if our model was not described with sufficient clarity. Our results suggest that both active invaginations of joint cells, through activation of Rho1 by Dysf, and proliferation in the interjoint regions promote the "puff-up" of the epithelium that generates the characteristic folding. To prove this, we have carefully measured tissue height as an indicator of cell behaviors during the formation of the tarsal 4/5-fold both in fixed tissues and in vivo (Fig. 4A-C, S5 Fig and movie 1). We found that while the height of the joint fold gradually decreases, the height of the interjoint domain progressively increased. In the absence of Dysf (Fig. 6A-C),

© 2024. Published by The Company of Biologists under the terms of the Creative Commons Attribution License (https://creativecommons.org/licenses/by/4.0/).

both processes do not occur properly and cells at the joint domain cells do not decrease their apico-basal height and interjoint cells do not elongate. Blocking cell proliferation prevents the compression of the tissue and the "puff up" of the interjoint cells while joint cells are not able to properly induce Rho1 activity, apical constriction and F-actin accumulation (Fig. 3 and Fig. 4D-E). These data demonstrate that there is active epithelial invagination in the joint region promoted by Dysf, which is further reinforced by the tissue compression forces such as those ones generated by cell proliferation (Fig. 4D-E) and by the extracellular matrix (Fig. 5).

In the new experiments presented in the revised version, we further tested the role of cell proliferation in fold induction. First, we ectopically expressed Dysf in the wing disc and investigated its ability to induce a fold in a proliferating tissue and in the absence of cell division. Our results demonstrate that fold induction by Dysf depends on cell proliferation, as transiently blocking cell division in the entire disc using a temperature-sensitive allele of Cdk1 prevents the formation of ectopic folds and the accumulation of F-actin (dppGal80>dysf, $Cdk1^{null/E1-24}$, Fig. 6D-F). However, blocking cell proliferation specifically in cells expressing dysf (dppGal80>dysf, Cdk1-RNAi, or E2f1-RNAi) has a minimal effect on the ability to induce a fold (Fig. S8). Second, we tested whether an increase in cell proliferation in the wing disc is sufficient to induce folding. In the previous version of the paper, we failed to observe the formation of folds; however, a more careful re-examination of the images and new experiments confirmed that this was indeed the case (Fig. 7D and E).

These results suggests that cell proliferation at a tissue-wide level is required for the induction of the folds, as we have described in the leg. These new results, which were not included in the previous version of the paper have been added in the new version.

2) Another alternative explanation/hypothesis for the presented data comes from the observation that there is a rather extensive change in the overall shape of the leg disc. Based on the images presented in the manuscript, it appears that the radius of dysf expressing cell domain shrinks, especially in case of ta1 (Fig. 1 B, C). In such a scenario, it seems also possible that cells intercalate within this domain, and perhaps also in other domains. Such intercalation could also lead to tissue expansion, as the same number of cells try to fit into a smaller domain, and could explain the domain folding. Thus, cell intercalation within inter-joint regions could also lead to the "puffing-up" of the inter-joint regions. The authors may want to consider such mechanisms as an alternative to cell divisions, and they also may want to comment on whether/how much intercalation could in principle contribute to the formation of epithelial folds.

We thank the reviewer for this comment. We agree that during the transition between late third instar and prepupae the leg begins to elongate along the proximo-distal axis, changing the overall shape of the leg. Cell intercalation has been reported to contribute to leg elongation and may influence in the formation of the epithelial folds. We have incorporated these possibilities in the discussion as factors that could also increase tissue compression besides cell proliferation. However, while cell intercalation during the evagination of prepupal leg discs has been documented (Taylor and Adler, Dev Bio 2008), our observations reveal that fold induction becomes apparent in mid-third instar leg discs, at least 24 hours earlier. It will be interesting to explore the contribution of cell intercalation to leg morphogenesis, however it is challenging because genetically interfering with factors that could disrupt it (myosin inhibition for example) also blocks cell proliferation and have a strong effect on the cytoskeleton.

3) It appears that the majority of proliferation occurs prior to formation of the epithelial folds, as the folds themselves are most apparent during pupal stages. It is, however, not clear how authors exclude the possibility that the fold formation is a result of change in tissue dimensions, rather than proliferation. For instance, the authors need to exclude that tissue extrinsic compression from, say, the dynamics in the peripodial membrane, causes the fold formation. In case of such extrinsic stresses, we can also have folds or no folds depending on how many cells existed at the larval stage, before the compressive forces were applied during pupal stage. In this sense, cell proliferation might indeed play a role in fold formation, though only indirectly, as the folds might not form in the absence of extrinsic compression despite prior cell proliferation. It would be helpful if the authors could clarify this point.

© 2024. Published by The Company of Biologists under the terms of the Creative Commons Attribution License (https://creativecommons.org/licenses/by/4.0/).

We apologize if the information in the paper was not sufficiently clear. Our results indicate that cell proliferation, through the progressive accumulation of cells, generates compressive forces that drives the folding of the epithelium at specific locations in the leg disc. As illustrated in Fig. 1B-D the onset of folding initiation coincides with a period of active proliferation during the third instar stage leg disc. Newly included images of the third instar leg discs (Fig. 1D) clearly depict these folds during this developmental stage. However, these folds are more clearly visible at the prepupal stage (Fig. 1E). We agree that the immediate consequence of reducing cell proliferation is its effect on tissue dimensions that impact on the generation of the compressive forces that are required to initiate epithelial folding at specific positions. Reducing cell proliferation prevents the formation of the tarsal epithelial folds in the leg disc, however these phenotypes are much easier to visualize and quantify at prepupal stages.
Besides cell proliferation, other elements such as the peripodial membrane and the ECM could contribute to generate the compressive forces required for epithelial folding in the leg. In this paper we have addressed the role of the basement membrane (Fig. 5), however, manipulating the peripodial membrane is a more difficult task.
To investigate the role of the peripodial membrane in the formation of tarsal epithelial folds, we genetically ablated it using a specific Gal4 line (apontic-Gal4) in combination with the tub-Gal80ts system to temporally express the proapoptotic gene hid in these cells. Although preliminary, our findings indicate that the genetic ablation of the peripodial membrane cells does not significantly affect the formation of tarsal folds. However, it does result in severly deformed and prematurely elongated legs (see Fig. R1 in the PDF file). We have chosen not to include this data as we intend to explore it in more detail in the near future.

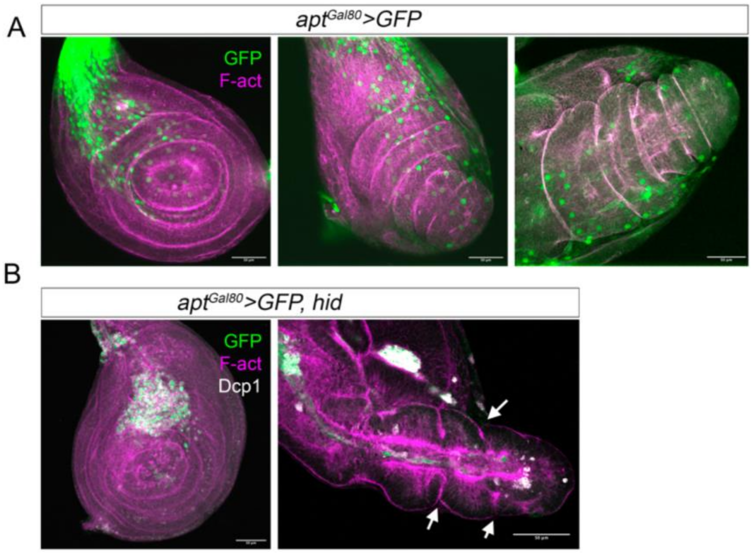

***Figure R1**: A) Expression of the apt-Gal4, tub-Gal80ts, UAS-GFP (apt^{Gal80}>GFP) in the peripodial membrane. B) Temporarily expression of the proapoptotic gene hid for 24 hrs in the peripodial membrane cells (apt^{Gal80}>GFP, hid) effectively induces cell death as visualized by the effector caspase Dcp1. Note that the leg is deformed although tarsal joints are formed.*

4) Assuming the authors can demonstrate that active indentation at the leg segments results in formation of folds (see point 1), the term ‘buckling’ still does not seem to be appropriate. The authors see that leg disc (and also wing disc) formation in Drosophila requires cell division in the disc itself. They interpret the need for cell division as the need for tissue expansion, which builds up compressive stress that eventually helps the disc to “fold up” in a stereotypical manner. From the manuscript, it reads such that the position of the “folds” are determined by genetic pre-patterning, and that folds never appear outside of the area where they are supposed to form. This phenomenon might better be called something like ‘compression-aided folding’, since buckling is typically described as a rather sudden and spatially stochastic event of material property failure. For this, data on dynamics of the process is required, which is admittedly difficult to achieve in fixed tissue. Additionally, leg/wing disc development occurs on rather long time scales, indicating that the build-up of

© 2024. Published by The Company of Biologists under the terms of the Creative Commons Attribution License (https://creativecommons.org/licenses/by/4.0/).

compressive stresses is rather slow, and would also lead to a slow relaxation of the same through gradual tissue deformation and remodeling. To give a few examples to explain this sentiment:

4.a) p.4 The authors say: "From mid-third instar larval stage to prepupa, we observed a sequential accumulation of F-actin in ring-like structures at the folding points, which ultimately form the joints separating the leg segments in the adult stage (Fig. 1A)".

To get a sense of the dynamics here: how long does this developmental window last? These are hours, aren't they? It would be helpful to clarify this so that the reader understands that the terms "folding", "apical constriction", "buckling", etc run on a different time scale. The authors do a good job of introducing these processes in various epithelial contexts in the introduction section, but they also need to highlight the differences in the time scales.

We agree with the referee that the term "buckling" may not be the most accurate word to describe the stereotypical folding of the leg epithelium. We have amended the text accordingly. The sequential accumulation of F-actin in the folding points refers to the progressive appearance of the different proximo-distal (PD) folds in the leg, a process that takes approximately 48hrs (from early third instar until prepupae). Specifically, the formation of a particular fold such as the ta4/ta5 fold last around 3-5hrs as is described in Fig. 4A-C and the live imaging movie. We have generated live imaging of the formation of this particular tarsal joint to illustrate not only the dynamics of fold formation but also to measure, as we did for fixed tissues, how the joint cells apically constrict and reduce their height while interjoint cells increase it in response to tissue compression (see movie 1 and S5 Fig).

4.b) p.6 The authors say: "We observed that posterior cells at the presumptive joint fold failed to properly constrict apically and show significantly less apical F-actin than control anterior cells (Fig. 3A, B and E)."

Similar to the point above, it is difficult to see apical constriction being such a slow process, especially since it has been described extensively in the embryo context, where it occurs at a time scale of seconds to few minutes.

Our results indicate that compressive forces that build up during imaginal disc development and during the first hours of prepupae development contribute with Dysf to generate a fold. Apical constriction and cell shortening at the joint cells due to tissue compression combined with Dysf-induced Rho1 and apico-basal elongation of interjoint cells promote the folding of the epithelium. We agree that apical constriction itself can proceed faster than fold formation. Specifically, the formation of the tarsal 4/5 folds takes around 3-5 hours, while the completion of all the different PD joint folds present in a leg is a longer process as indicated above. We have not measured the dynamics of apical constriction of individual cells during fold formation, but it might be similar to what's observed in the embryo (seconds to a few minutes).

In our experiments, we blocked cell proliferation for 48hrs to ensure that we were affecting all of the tarsal joints from their initiation to their completion and analyzed the phenotypes in prepupal legs. Therefore, the consequences of reducing proliferation on apical constriction, F-actin accumulation, and fold formation were analyzed at a stage when folds are already formed in the control compartment.

4.c) p.8, end of second paragraph: "These results suggest that compressive forces generated by cell proliferation promote the upward buckling of the interjoint epithelium and thus contribute to the formation of the tarsal folds"

"Buckling" would be the case, if, w/o actin and MyoII, there is still deformation. But that does not seem to be the case. While buckling may be defined as a passive deformation due to tissue extrinsic factors (forces), the accumulation of F-actin and MyoII that we see in the manuscript rather indicates active and tissue intrinsic causes. Also, the position of the folding remains stereotypical and is not random, arguing against a stochastic failure of material properties (which is buckling).

© 2024. Published by The Company of Biologists under the terms of the Creative Commons Attribution License (https://creativecommons.org/licenses/by/4.0/).

We agree with the reviewer and changed the text accordingly.

4.d) p.8, second-last sentence: "These observations suggest that an increase in tissue compression resulting from cell proliferation increase epithelial folding at specific positions."

In contrast to the sentence mentioned in 4.c, this statement is much less contentious. Maybe the authors could generally shift the narrative to be consistent with this phrasing, and away from use of the term 'bucking'.

Yes, we agree with the referee and changed the manuscript to remove the term buckling.

5) The authors imply a causal relationship between cell height and tissue compression, see p.14, first new paragraph on the page: "A detailed analysis of the phenotypes resulting from reduced cell numbers uncovers a previously unanticipated behavior of the interjoint cells - their ability to increase in height in response to tissue compression.
Based on the presented data it is difficult to conclude that this is really the case. The implied causal relationship between cell height and tissue compression would require support from live imaging. The authors could either rephrase this, or perform additional experiments to exclude the possibility that change in cell height facilitates tissue deformations - showing this through simulations of leg epithelial folding is not sufficient.

As suggested by the reviewer we have performed live imaging of ta4/ta5 joint fold formation to quantify the dynamics of changes in joint and interjoint tissue dimensions. In accordance with our fixed tissue measurements, we found that joint cells decrease their apico-basal height, whereas interjoint cells increase it (S5 Fig and movie). Nevertheless, these findings fail to discern the causal connection between tissue compression and cell height in the course of joint formation. To address this limitation, our experiments in which we inhibit cell proliferation or eliminate the ECM provide more conclusive evidence. By diminishing tissue compression, we directly influence the apico-basal height of interjoint cells (see Fig. 4A-E and Fig. 5B-C).

Altogether, our data points to a causal relationship between an increase on tissue compression (due to the accumulation of cells in a confined space) and the tissue deformations that leads to the formation of tarsal folds at specific locations dictated by Notch/Dysf. We have modified the text, as indicated above, to also highlight the contribution that invaginated cells could have on the elongation of interjoint cells.

6) Studying epithelial morphogenesis in leg disc formation is, without question, challenging. To highlight the efforts and let the reader appreciate the insights gained through this model, the manuscript would benefit from a better description of inherent limitations in the experimental approach and corresponding caveats in the conclusions. For instance, it does not become clear for a non-expert reader why the authors would not use live imaging data, in spite of being able to express fluorescently labeled proteins. At the same time, live imaging immediately comes to mind as a possibility to exclude various hypothetical possibilities, and strengthen the causal relationships (see alternate hypotheses above). Based on the citations of articles previously authored by some of the authors in this study, the authors have extensive experience in working with leg discs, and readers would benefit from a description of experimental limitations from the authors perspective. Again, as an example for the case of live imaging: if this is technically challenging, then it would help if authors mention it and cite a few precedents.

We agree with the referee that live imaging of the epithelial folding process could provide very valuable information to understand the dynamics and cell behaviors during this process. However, as the referee points out, live imaging of the leg disc is technically challenging, especially during the transition from a relatively flat epithelium of an early third instar disc to the prepupal disc. For this reason, we have live-imaged the formation of the ta4/ta5 fold as it is the last one to be formed. We have measured the dimensions of the epithelial cells as we have done for the fixed tissue obtaining similar results. Unfortunately, the cellular resolution of the movies is not as good as in the fixed tissues. We have included these new data and movie in Fig. S5 and movie 1.

© 2024. Published by The Company of Biologists under the terms of the Creative Commons Attribution License (https://creativecommons.org/licenses/by/4.0/).

Minor concerns:

1) It is a bit challenging to raise specific points without line numbers in the manuscript.

We apologize for any inconvenience this may have caused. Some journals do not accept them.

2) Please switch to color-blind-friendly pseudo-color combinations in colored image panels.

We have reviewed all the figures and adjusted them to make them accessible to colorblind individuals.

3) Please add scale bars on all representative images. Needless to say, separate scale bars are not required when different channels of the same image are displayed, or if it is obvious that various images are acquired in the same region with the same dimensions. But, this is not the case in a lot of images. Since no scale bar was shown, we had to make assumptions about the scale in the following figures/panels: Fig. 1B, C, Fig. 3, Fig. 4, Fig. 6A, B, D (cross-section views), Fig. 7, and Supplementary Fig. 1-5. Dimensions are relatively easy to gauge when the reader is experienced in looking at microscopy images, but still: please do not force the reader to make assumptions about the scale in the image panels.

We apologize for forgetting this important detail that we have incorporated in all the figures.

4) p.4 The authors say: "From mid-third instar larval stage to prepupa, we observed a sequential accumulation of F-actin in ring-like structures at the folding points, which ultimately form the joints separating the leg segments in the adult stage (Fig. 1A)".

It may be better to say this more carefully: increased phalloidin suggests accumulation of actin. Also, please rephrase to highlight that this is a "tissue-scale" ring structure, which is rather distinct from "cellular-scale" F-actin rings seen in cytokinesis.

We have modified the text accordingly, and also included better images (see Fig. 1) to reflect that the accumulation of F-actin indicates the formation of the fold joints in third instar leg discs.

5) p.4 The authors say: "Dividing cells are found throughout the leg imaginal discs with no particular cell division pattern and their number progressively decreases during early prepupal stages (Fig. 1A and S1 Fig)." It would help if the authors support their quantitative statement about the decreasing number of dividing cells, with quantification of number of dividing cells. What is the fraction of dividing cells? Is it the absolute number or the fraction of dividing cells that's more relevant? Is the number/fraction of dividing cells similar in anterior vs. posterior compartment? How about joint vs inter-joint regions? How would these differences/similarities affect the outcome of the hypotheses about the involvement of cell proliferation on fold formation?

We have quantified the number of dividing cells during the formation of the epithelial folds (from early third instar to 3- 5 hrs APF) and incorporated the data in S1 Fig.

Using different cell proliferation markers such as pH3 and EdU, we have not found any differences between Anterior and Posterior compartments nor interjoint/joint domains.

**6) p.5 in the same paragraph the authors say, "However, we did not observe a reduction in the mitotic index in the wing disc, calculated as the number of pH3-positive cells per area, suggesting that cells are proliferating at a slower rate (S2 Fig)."
This statement about the wing disk appears somewhat out-of-the-blue. It is unclear why the student's t-test was performed in S2 Fig. D when the data in E2f1 RNAi is bimodal. Here, a non-parametric test is recommended. Also, the definition of mitotic index could also be included in the methods, if space permits.**

Due to space limits in the text we decided to remove this supplementary figure as it does not add

© 2024. Published by The Company of Biologists under the terms of the Creative Commons Attribution License (https://creativecommons.org/licenses/by/4.0/).

to the work. Instead, we have included the quantification of the number of cells after reducing cell proliferation by the downregulation of E2f1. This data is now included in Fig. S2.

7) p.8 authors say, "Next, we analyzed the height of interjoint and joint cells after reducing cell proliferation by expressing the E2f1-RNAi line in the posterior compartment, and compared it with the anterior control in 3 hrs APF leg discs."

It is not quite clear where to find the reference for the quantification in comparing anterior and posterior compartment. For the quantifications discussed in the results section 'Tissue compression generated by cell proliferation is necessary for leg epithelial folding', presented in Fig.4 and S5, it isn't clear when the measurements are made in the anterior compartment vs. in the posterior compartment. This needs to be clearly explained. If the control condition mentioned in the figure is indeed measurements in the anterior compartment, then it is unclear why the measurements were not compared between a purely genetic outcross control vs. perturbations, as the indentation depth seems to be different between anterior and posterior sides of the indentation even in control leg disks.

We apologize for the confusion and we have changed the text to better reflect how the comparisons were made. We consider that comparing the formation of the tarsal folds in the posterior compartment (experimental) to the anterior compartment (control) is the most accurate method of measuring the effect of cell proliferation on tissue height and folds.
This method is particularly useful as it allows for comparison with an internal control within the same disc, enabling the normalization for slight developmental stage differences that could interfere with the quantifications. To reinforce our findings, we cross-validated our experimental data—expressing the indicated transgene in the posterior compartment with hh-Gal4—with an experimental control using a RNAi against cherry. Importantly, both comparisons of tissue height in the posterior compartments yielded identical results (see Fig. R2 in the PDF file).

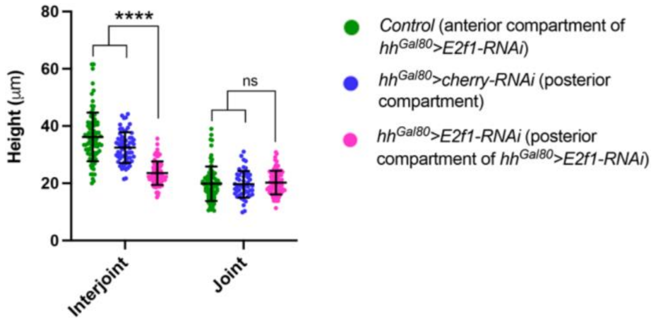

Figure R2: *Quantification of tissue height in the interjoint and joint domains of the genotypes indicated. Note that after reducing proliferation in the posterior compartment, interjoint cells decrease their height when compared to cell from the anterior control compartment or to cells from the posterior compartment of hh^{Gal80}>cherry-RNAi.*

The possible differences in the folding that might be observed between the anterior and posterior compartments in a control disc are likely to be due to that specific image and focal plane and are not representative of the entire leg. Prepupal legs, which are cylindrical, could give the impression of slight differences in folding between compartments in sagittal sections. However, to ensure the accuracy in our quantifications, we were very cautious and captured multiple Z-images from the same prepupal leg and consistently measured tissue dimensions by selecting the most appropriate Z-section for each compartment.

Reviewer 2 Advance Summary and Potential Significance to Field:
The manuscript addresses the role of cell proliferation in driving the formation of folds during tissue morphogenesis using the tarsal fold in the Drosophila leg as a model. The authors find that global proliferation results in compressive forces. These forces coordinate with Notch signaling to result in buckling or folding of the tissue at regions where Rho1 is active in a Notch-dependent manner. While the idea that global cell proliferation under constraint drives tissue buckling is not novel, coupling this finding with a (Notch) signaling pattern to address the

© 2024. Published by The Company of Biologists under the terms of the Creative Commons Attribution License (https://creativecommons.org/licenses/by/4.0/).

stereotypic nature of (tarsal) tissue folds is a significant advance in the field. The manuscript is well-written, with compelling and elegant experiments. I also appreciate the exhaustive nature of ruling out key mechanisms and testing the applicability of mechanisms in another (wing) tissue.

We thank the referee for the positive comments and suggestions that we carefully addressed in this revised manuscript.

Reviewer 2 Comments for the Author:
Major comments for revision:

1) All images use a red-green combination. Please use a color combination that is accessible for folks with color blindness. A green-magenta combination works well, in my opinion.

We agree with the suggestion and changed all the figures to make them accessible to colorblind individuals.

2) Figure 3: Rho 1 accumulation in folds and lack thereof in proliferation-deficient and, thereby, fold-deficient tissue is a correlation, not causation. The authors can try to down-regulate Rho1 activity in the folds to examine its role when proliferation is unaffected. This maybe tricky if Rho1 is also involved in proliferation but the experiment may still work if Rho1 is knocked down only in the joint cells.

The role of Rho1 activity during tarsal fold formation and its regulation by Dysf was studied in our previous paper (Cordoba and Estella, PloS Genetics 2018). In that paper we described that Dysf regulates Rho1 activity at the tarsal epithelial folds and that Rho1 function is required for fold and adult joint formation. Moreover, we also investigated the role of the Rho1 effectors Rok, Drak and Sqh.

In the present work we show that reducing cell proliferation lowers Rho1 activity levels without affecting dysf expression. This result suggests that that proliferation-induced tissue compression reinforces Rho1 activation and apical constriction prompted by Dysf.

To follow the referee suggestion, we specifically downregulate Rho1 function in the folds by expressing a dominant- negative form (Rho1^{DN}) using the dsyf-Gal4 line. In line with our previous results, we observed that the folds were not formed, however the integrity of the epithelium was compromised in some cells (Fig. R3 in the PDF file). We also noticed a strong effect on tissue size probably due to the contribution of Rho1 in cell division and cell survival.

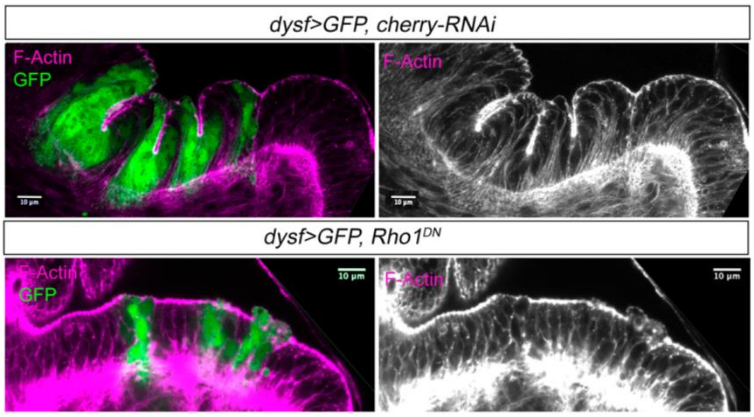

Figure R3: *Prepupal leg imaginal discs from the genotypes indicated and stained for F- actin. Note the absence of epithelial folds and the effects on tissue integrity and size after expressing the dominant negative of Rho1 in the tarsal folds.*

3) Fig 4: How the authors measured cell height needs to be clarified. It seems inaccurate if the double-headed straight/perpendicular arrow is trying to follow F-Actin. I couldn't find it in the

© 2024. Published by The Company of Biologists under the terms of the Creative Commons Attribution License (https://creativecommons.org/licenses/by/4.0/).

methods section either. One way to resolve this is by salt pepper labeling of the cell membrane to accurately measure where the boundary of the cells is- the cell's height may be at an angle from the base following the bends (rather than orthogonal from the base). Inferring compressive forces from cell height is also unclear - is the argument that taller cells are compressed while the volume conserved? Apical constriction in joint cells can also create "compressive forces," resulting in the expansion of interjoint cells (rather than cell proliferation alone).

We apologize for the lack of clarity. We have modified the text to reflect how the measurements were made. We agree that to obtain an accurate measurements of cell height it is necessary to label individual cells. However, as the imaginal disc epithelium is a monolayer of pseudostratified cells the height of the tissue could be extrapolated to the relative height of a cell in a specific domain. To illustrate this, we have generated single cell clones to visualize cell shapes during fold formation (see Fig. R4 in the PDF file*). These results have been also incorporated in the revised paper (Fig. S5). Anyway, we have modified the text to indicate that our measurements refer to the dimensions of the epithelium and not of the cells. In the Materials and Methods section, we have also clarified how these measurements were carried out*
"Tissue height was quantified using sagittal and cross-sectional images from prepupal leg and imaginal discs, respectively and stained with phalloidin. Cross-sectional images from wing imaginal discs were obtained by Fiji from multiple Z-stacks that cover the whole disc. As the main epithelium of the wing and leg imaginal discs is formed by a monolayer of cells, the height of the tissue could be extrapolated to the relative height of a cell in that specific domain. To do this in the leg disc, we measure the distance from the basement membrane of the epithelium to the highest point in the interjoint domain (proximal to dysf) and to the lowest point in the joint domain (distal to dysf) (see Fig. 4A). In the wing disc we measured the distance from the base of the epithelium to the deepest point in the region of interest or fold (see Fig. 6D).

We also agree that compressive forces generated by apical constricting cells at the joint domain could also result in the apico-basal expansion of interjoint cells. We have incorporated this possibility in the discussion.

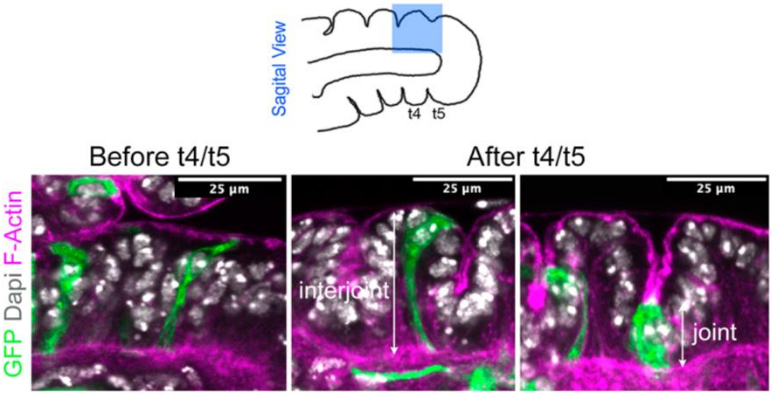

Figure R4: *Single-cell mCD8::GFP- labeled clones were observed before and after the formation of the 4/5 tarsal fold. Notice how the interjoint cells increase their apico-basal height, while joint cells decrease it. Additionally, the pseudostratified epithelium undergoes a transition from a complex arrangement of nuclei to a simpler one, with all nuclei aligning.*

4) Fig 6A: Is cell proliferation normal in dysf RNAi? If yes, it supports the result in the posterior part, where dysf is downregulated, the tissue is wavy/buckling, albeit abnormally or in a non-patterned way. Relatedly, can the authors express a CA form of Rho in a spatially controlled manner to rescue the dysf RNAi phenotype?

We have analyzed cell proliferation (mitotic index) after knocking-down Dysf in the distal domain (Dll>dysf-RNAi) and compared it to a control situation (Dll>cherry-RNAi). As illustrated in Fig. R5 in the PDF file*, no changes were observed in the mitotic index, calculated as the number of pH3-positive cells per area, were observed. These results have been included in the revised*

© 2024. Published by The Company of Biologists under the terms of the Creative Commons Attribution License (https://creativecommons.org/licenses/by/4.0/).

manuscript in Fig. S7.
In a similar experiment as suggested by the referee, in our previous paper (Cordoba and Estella, PloS Genetics 2018), we demonstrated that the expression of Rho1 and Rho1 downstream effectors is sufficient to form ectopic folds in the wing disc where there is no Dysf. Similarly, ectopic expression of Dysf is also able to induce ectopic folds in the wing.

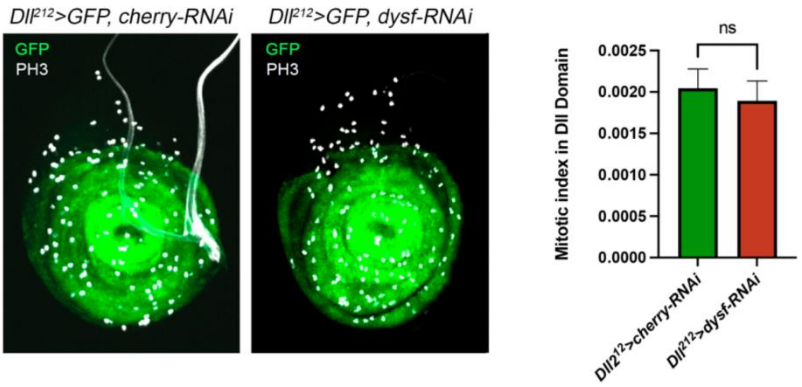

Figure R5: *Downregulation of Dysf in the distal domain of the leg (Dll>GFP, dysf-RNAi) doesn't affect the proliferation of the cells measured as ratio of pH3 positive cells per area in the Dll domain (green) when compared to the control (Dll>GFP, cherry-RNAi).*

Minor:

1) Fig 1C: It needs to be clarified what is apical and basal. It becomes clear with the BM staining later. Please annotate this figure.

We have indicated in Fig. 1E the location of apical and basal.

2) Fig 2F: Rbf280 does not look like control in the anterior section, and CyCE tissue looks bigger- can be discussed further.

The possible differences in the folding that could be observed in a particular image are likely attributable to the plane and the relative position of the prepupal leg in the image. Prepupal legs, which are cylindrical, could give the impression of slight differences in folding between compartments or between images in sagittal sections. In some images, the folding could be more advanced or more compacted than in others. However, to ensure the accuracy of our quantifications, we were very careful to capture multiple Z-images of the same prepupal leg and consistently measured tissue dimensions by selecting the most appropriate Z-section for each measurement.

3) S4A: Are there fewer folds overall? Even in control?

The folding pattern observed is normal and within the variability observed in wild type animals. As discussed above, sagittal sections, could create an impression of slight folding differences between images.

4) Fig 6 D: Measuring cell height with orthogonal lines does not seem accurate as the phalloidin staining is at an angle following the bending of the cells (see major comment 3).

Please refer to our reply of major comment 3.

5) Scale bars need to be included in several figures. Please add them everywhere.

We apologize for forgetting this important detail that we have incorporated in all the figures.

Reviewer 3 Advance Summary and Potential Significance to Field:

© 2024. Published by The Company of Biologists under the terms of the Creative Commons Attribution License (https://creativecommons.org/licenses/by/4.0/).

In this manuscript, Rodriguez and colleagues investigate the role of Notch signaling and cell proliferation in the formation of epithelial folds. Using the Drosophila leg as a model, they find that effective formation of leg tarsal folds requires a combination of proliferation within the epithelium together with Notch signaling to specify the location of the folds; specifically, the Notch target dysfusion is responsible for producing targeted action of actomyosin within the joint cells to shorten their apical-basal length and causing their apical constriction. The authors propose a mechanistic model requiring the coordinated action of proliferation (within the entire tissue, not merely the joint cells themselves), which creates tissue-wide compression forces, together with dysfusion-dependent control of the height and apical constriction of joint cells, which together produce epithelial buckling at the appropriate locations.

The paper is overall well written, and the authors make a compelling case in the first part of their narrative that (1) a reduction of proliferation results in a flattened epithelium and a loss of tarsal folds (while Notch activity and localization are preserved), and that this effect requires a reduction of global proliferation (both interjoint and joint cells), rather than merely that of joint cells; (2) that in proliferation-deficient cells, Rho1 activity and apical constriction are reduced.

The authors go on to propose that proliferation creates tissue compression, which is measured experimentally through the height increase of the interjoint cells with a concomitant height decrease of the joint cells; the generation of the compression forces requires an intact ECM. In addition, the authors propose that Dysf is directly responsible for Rho1-mediated apical-basal shortening of the joint cells (as well as apical constriction), and follow up with a straightforward computational finite-element model that recapitulates the distinct roles of proliferation (leading to compression) and Dysf-dependent apico-basal height constraint in generating epithelial folding.

In this reviewer's opinion, a strength of this paper was its compelling narrative and good logical flow. One experimental detail that I particularly appreciated was the approach of specifically knocking down the function of specific genes in the posterior compartment, so that control (anterior) and knockdown (posterior) could be directly compared in the same animal. In addition, the mechanistic model - the generation of controlled buckles through a combination of planar compression forces with locally controlled apico-basal asymmetries is new in this particular system and of general interest to the morphogenesis field.

However, I was not as convinced by the data in the second part of the manuscript as in the first part, and I thought that the analysis lacked resolution and rigor in some areas that were critical to making some of the authors' key points.

We would like to thank the reviewer for their positive comments and useful suggestions and criticisms. We have addressed these in the revised manuscript and provided responses point by point below.

Reviewer 3 Comments for the Author:
MAJOR:
(1) The authors make the point more than once that Drosophila leg development is merely one model system for epithelial folding, and that insight into the physical mechanism of epithelial folding in this system will potentially transfer to other systems. While I completely agree, I think it's really unfortunate that the current manuscript is not really written with those 'transfer' readers in mind: I think the current images are hard to understand for any readers other than those who already have a lot of experience with looking at images of Drosophila leg imaginal discs and tarsal folds. In this reviewer's opinion, the introduction section of the manuscript would benefit enormously from a schematic like the one in Figure 8H that shows (a) the layout of the region of the interest within the organism, and (b) especially critically, shows a conceptual drawing of the architecture of the individual cells (with cell-cell junctions and nuclei indicated) within the epithelial tissue, because the cell-cell junctions are very hard to see in the experimental images.

We acknowledge the reviewer for its suggestion and therefore included in Fig. 1A a drawing of a

© 2024. Published by The Company of Biologists under the terms of the Creative Commons Attribution License (https://creativecommons.org/licenses/by/4.0/).

third-stage larva with the imaginal leg disc colored in magenta and the development of the disc up to the adult stage. We also included a cartoon of the epithelial folds as the one included in Fig. 8H. The architecture of the epithelium with the cell-cell junctions can be easily visualized with the baso-lateral protein Disc-large (Dlg) as indicated in Fig. 4D.

(2) This reviewer finds it disappointing that the entire argument about the relationship between proliferation and compression hinges on measuring a single one-dimensional (and very reductive) parameter, namely the height of the interjoint vs joint cells. Both the scope and Methods description of the current image analysis is very sparse. The authors' mechanistic model about the deformation of the tissue through a combination of proliferative compression with mechanical constraint really begs for live imaging (where individual animals are tracked over sufficient time periods to observe the gradual deformation of the different cell populations with concomitant recruitment of actomyosin and associated tissue bending/folding). I appreciate that performing live imaging experiments may not be entirely feasible, or the authors may plan to do this for their next major project/paper on this topic. However, if the authors' narrative (and the computational model) hinges on the generation of compressive stress, and if they want to make this point convincingly based on immunostaining and fixed images alone, then I would really encourage them to dig deeper into their current data to describe and analyze the organization and re-organization of the cellular architecture during fold formation, and make a convincing attempt at a more robust 2-dimensional description.

We apologize for the lack of clarity. We have modified the text to reflect how the image analysis and quantifications were made. Tissue height was quantified using sagittal and cross-sectional images of prepupal leg and imaginal discs, respectively and stained with phalloidin. Cross-sectional images of wing imaginal discs were obtained by Fiji from multiple Z-stacks that cover the whole disc. As the main epithelium of the wing and leg imaginal discs is formed by a monolayer of cells, the height of the tissue could be extrapolated to the relative height of a cell in that specific domain. To do this in the leg disc, we measure the distance from the basement membrane of the epithelium to the highest point in the interjoint domain (proximal to dysf) and to the lowest point in the joint domain (distal to dysf) (see Fig. 4A). In the wing disc we measure the distance from the base of the epithelium to the lowest point in the region of interest or fold (see Fig. 6D).
Please refer to Fig.R6 below for more detail on how measurements were made.

We thank the reviewer for its comment. Ideally, live imaging with appropriate markers to label cell membranes, nuclei, actomyosin dynamics and stress sensors would provide very valuable information. However, this is extremely challenging as the leg imaginal disc changes in shape very fast during the formation of the folds, from a relatively flat epithelium in early third instar to the everted 3D prepupal leg. Furthermore, the level of detail required to perform the quantifications we have carried out in fixed tissue is not easily attainable for in vivo images. However, as suggested by the reviewer we have attempted to perform live imaging during the formation of the epithelial folds. We decided to focus on the ta4/ta5 epithelial fold as it is the last one to be formed. Consistent with our previous observations using fixed tissues, our live imaging reveals that the formation of the fold involves not only apical constriction and a reduction in apico-basal size of the cells within the joint, but also an increase in height of the cells in the interjoint region. Notably, these cell shape changes are not observed upon reducing cell proliferation, resulting in the absence of the fold, as evident from our quantifications in the fixed tissues depicted in Fig. 4D and E.
We have included this data as S5 Fig and movie 1. In the future we will try to improve our live-image set-up to analyze other parameters during wild-type and cell proliferation reduced situations. These experiments will follow up the important observations that we have described in this paper.

Some information that would have helped me interpret and contextualize the current analysis: Can you show explicitly (e.g. by counting nuclei, or segmenting cell-cell junctions) from your current images that cell numbers increase in specific areas over time due to proliferation? Can you show explicitly how these cell numbers are reduced in the phenotypes with reduced proliferation? Can you measure how the total volume of the epithelial sheet changes over these time scales (and thus how cell volumes change during proliferation)? Can

© 2024. Published by The Company of Biologists under the terms of the Creative Commons Attribution License (https://creativecommons.org/licenses/by/4.0/).

you measure - or at least show in processed or segmented image where the cell-cell boundaries are clearly visible - how the distribution of cell volumes (in the apico-basal) direction changes over time? For example, it's a key feature of the current computational model (Figure 8) that interjoint cells expand apically and joint cells expand basally in panel B and C but not in panel D - yet in the accompanying experimental images in Figure 8B,C and D (which are supposed to represent these three different phenotypes) it is absolutely impossible to tell whether that's actually the case.

During the formation of the leg epithelial folds, a process that takes approximately 48 hrs from starting at mid-third instar until they are fully visible (4-5 hrs APF), the leg imaginal disc increases in cell number and size as can be observed in Fig. 1B. Using cell proliferation markers such as pH3 and EdU we found no differences between specific areas such as the interjoint/joint domains. Our data indicates that cell proliferation acts as a tissue-wide mechanism that helps to increase the tissue compression forces, which in coordination with the patterned activity provided by Notch/Dysf (Rho1 activation, apical constriction and F-actin accumulation) promotes the formation of the folds at specific positions along the proximo-distal axis.

We have analyzed how the reduction of cell proliferation in specific domains, such as the posterior compartment in the hh^{Gal80}>E2f1-RNAi experiment, affects the number of cells using a program developed in-house (Ledesma-Terrón, M et al 2021 bioRxiv 2021.06.25.449919; doi: https://doi.org/10.1101/2021.06.25.449919) that segments and localizes individual nuclei in a crowded three-dimensional confocal reconstruction. We found that the hh^{Gal80}>GFP, E2f1-RNAi experiment reduced the number of cells in the posterior compartment of the distal leg aprox. 35% compared to the control posterior compartment of hh^{Gal80}>GFP, cherry-RNAi legs. We have included these data in S2 Fig.
As the referee points out, one feature of the fold formation in the computational model is that in a wild type scenario, the apico-basal enlargement of interjoint cells occurs while joint cells expand basally. To clearly show the changes in cell shape in the apico-basal direction over time during fold formation, we have generated marked single cell clones before and after the formation of the ta4/ta5 fold. As illustrated in Fig. S5 and Fig. R6, we observed that joint cells decreased their apico-basal height while interjoint cells increased it. Measurements of tissue dimensions in both fixed and in vivo tissues have been included in the revised manuscript (Fig. 4A-C and S5 Fig and Movie 1).

An important consequence during the formation of the folds is that the epithelium changes from a pseudostratified morphology with nuclei located at different positions along the apico-basal axis to a simpler morphology with all the nuclei aligned and positioned at the apical side (see Fig. S5, Fig. 8B). This cell rearrangement likely is a consequence of the tissue compression forces generated by cell accumulation and the activity of Notch/Dysf, which promotes the shortening of joint cells. To better illustrate the shape changes after reducing cell proliferation or knocking-down Dysf we have labeled the cell membranes with Dlg and F-Act and separated the channels in Fig. 8B and Fig. R6 in the PDF file. After reducing cell proliferation or in the absence of Dysf, the epithelium is maintained as pseudostratified and no changes in cell shape such as apico-basal elongation of interjoin cells neither basal expansion of joint cells were observed. We have tried to segment these images but the ratio signal vs noise was very low and the location of the cells in different planes complicated this task.

© 2024. Published by The Company of Biologists under the terms of the Creative Commons Attribution License (https://creativecommons.org/licenses/by/4.0/).

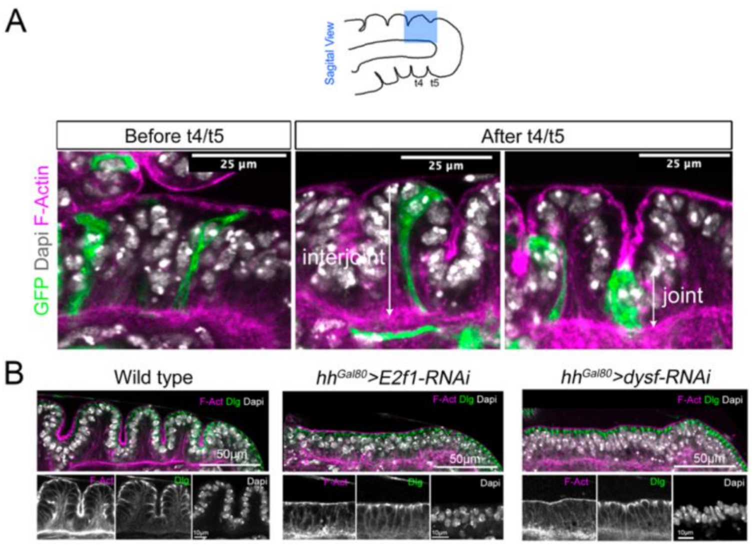

Figure R6: A) *Single-cell mCD8::GFP- labeled clones were observed before and after the formation of the 4/5 tarsal fold. Notice how the interjoint cells increase their apico-basal height, while joint cells decrease it. Additionally, the pseudostratified epithelium undergoes a transition from a complex arrangement of nuclei to a simpler one, with all nuclei aligning.*
B) Cell shape changes observed after reducing cell proliferation and after knocking down Dysf.

(3) If I understand correctly, the mechanistic model (incorporated into the computational model) has two required components: Force generation through compression - created by proliferation in combination with some mechanical constraints like the ECM adhesion - plus a programmed symmetry-breaking mechanism, which require dysfunction-dependent mechanical processes in the joint cells to produce the 'seeds' for the buckles. The authors' data seems to suggest that the symmetry-breaking mechanism has two components: One being the constraint (or even active reduction) of the height of the joint cells, the other being the apical constriction of the joint cells, both of which require dysf function and actomysin activity. Based on the results in Figure 3, do the authors have thoughts about whether one or both of these (height control and apical constriction) also require compression forces in order to activate - if so, how (e.g. maybe through mechanosensation of planar force components)? And if so, isn't that a key element that would need to be incorporated into the model? In addition, the computational model makes some key predictions (e.g. in absence of dysf function, we would expect cells to grow indiscriminately higher with proliferation; for increased overall proliferation, cells might start by growing indiscriminately higher with proliferation, and then buckling in random fashion), which are not reflected by experimental findings. I am quite supportive of the inclusion of the computational model, but if the model doesn't accurately reflect the empirical findings, how useful is it? Or is that the indication that some key element is missing that would reconcile those discrepancies?

Yes, the referee has understood it correctly. As illustrated in Fig. 3 and Fig. 4 both apical constriction and height shortening of joint cells depend on the compressive forces generated by cell proliferation and Notch/Dysf activity. We don't know what is the mechanism, but as the referee points out, a mechano-sensation of planar force components is clearly a possibility that would be interesting to explore in the future.

In the computational simulation, our purpose was to generate a conceptual model of the initial steps of epithelial fold formation where we could specifically study the interplay between tissue compression forces generated by cell proliferation (within the constraints of the ECM and peripodial membrane) and the activity of Notch/Dysf preventing the apico-basal elongation of joint cells. Since the main purpose of the model was to specifically test the role of the compressive forces generated by the accumulation of cells in the cell shape changes that follows the formation of the folds, we decided to not include the action of apical constriction and apico-basal height reduction observed in vivo in the joint cells. We believe that adding these additional features will ultimately result in a much more complex model where the interplay between

© 2024. Published by The Company of Biologists under the terms of the Creative Commons Attribution License (https://creativecommons.org/licenses/by/4.0/).

tissue compression and Notch activity will be obscured by the added complexity. In the model presented in Fig. 8, as cells proliferate and increase the density of the tissue, the blue cells (interjoint domain cells) start to grow apically compressing the apical domain of red cells. Including in the model active apical constriction and apico-basal height reduction, processes that are regulated by Dysf-induced Rho1, would not allow us to observe the effect of tissue compression on the apico-basal cell shape changes of joint cells. However, we agree that these elements play also very important roles as indicated by the absence of Dysf.

In the absence of Dysf, the folds are lost due to defects in Rho1 activation, F-actin accumulation and apical constriction at the joint cells. However, in this scenario, cell proliferation is normal (please refer to Fig. S7), and the tissue accommodates the same number of cells while maintaining the complex pseudostratified arrangement of the tissue. This occurs without the induction of random folding, either experimentally or in the simulation. In the computational model, in contrast to what happens in vivo, there is no restriction on the ability of cells to expand in the apico-basal axis, which is why cells are taller in the absence of Dysf than in the in vivo situation (Fig. 8D).

Second decision letter

MS ID#: DEVELOP/2023/202384

MS TITLE: Cell proliferation and Notch signaling coordinate the formation of epithelial folds in the Drosophila leg

AUTHORS: Alonso Rodriguez, David Foronda, Sergio Cordoba, Daniel Felipe-Cordero, Antonio Baonza, David Miguez, and Carlos Estella
ARTICLE TYPE: Research Article

I am happy to tell you that your manuscript has been accepted for publication in Development, pending our standard ethics checks.

Reviewer 1

Advance summary and potential significance to field

The present manuscript by Rodriguez et al. addresses the role of cell proliferation and notch signaling on the morphogenesis in leg discs during late larval and early pupal stages in Drosophila. It provides exciting insights into an understudied area of leg disc epithelial morphogenesis and highlights the role of non-cell autonomous contributions to local tissue folding. The manuscript provides an exciting contribution to the field and establishes the leg disk as a model system to understand epithelial folding as an interplay of genetic and mechanical cues.

Comments for the author

With their new, improved and revised version, the authors have undertaken impressive efforts to successfully address and resolve the issues that were raised. Congratulations on an exciting work and looking forward to seeing the manuscript published with Development.

Reviewer 2

Advance summary and potential significance to field

The concerns raised have been appropriately addressed by the authors. I have no further comments and recommend publication.

© 2024. Published by The Company of Biologists under the terms of the Creative Commons Attribution License (https://creativecommons.org/licenses/by/4.0/).

Comments for the author

The concerns raised have been appropriately addressed by the authors. I have no further comments and recommend publication.

Reviewer 3

Advance summary and potential significance to field

Using the Drosophila leg as a model, the authors find that effective formation of leg tarsal folds requires a combination of proliferation within the epithelium together with Notch signaling to specify the location of the folds; specifically, the Notch target dysfusion is responsible for producing targeted action of actomyosin within the joint cells to shorten their apical-basal length and causing their apical constriction. The authors propose a mechanistic model requiring the coordinated action of proliferation (within the entire tissue, not merely the joint cells themselves), which creates tissue-wide compression forces, together with dysfusion-dependent control of the height and apical constriction of joint cells, which together produce epithelial buckling at the appropriate locations.

Comments for the author

In this reviewer's opinion, the authors have adequately responded to reviewers' critiques and addressed the most important open questions.

© 2024. Published by The Company of Biologists under the terms of the Creative Commons Attribution License (https://creativecommons.org/licenses/by/4.0/).
